# A Comprehensive Overview of the Complex Role of Oxidative Stress in Aging, The Contributing Environmental Stressors and Emerging Antioxidant Therapeutic Interventions

**DOI:** 10.3389/fnagi.2022.827900

**Published:** 2022-06-13

**Authors:** Evripides Iakovou, Malamati Kourti

**Affiliations:** ^1^Department of Life Sciences, European University Cyprus, Nicosia, Cyprus; ^2^Angiogenesis and Cancer Drug Discovery Group, Basic and Translational Cancer Research Center, Department of Life Sciences, European University Cyprus, Nicosia, Cyprus

**Keywords:** aging, oxidative stress, antioxidants, envionmental pollution, mitochondria, reactive oxygen species, pathogenesis of aging

## Abstract

**Introduction:**

Aging is a normal, inevitable, irreversible, and progressive process which is driven by internal and external factors. Oxidative stress, that is the imbalance between prooxidant and antioxidant molecules favoring the first, plays a key role in the pathophysiology of aging and comprises one of the molecular mechanisms underlying age-related diseases. However, the oxidative stress theory of aging has not been successfully proven in all animal models studying lifespan, meaning that altering oxidative stress/antioxidant defense systems did not always lead to a prolonged lifespan, as expected. On the other hand, animal models of age-related pathological phenotypes showed a well-correlated relationship with the levels of prooxidant molecules. Therefore, it seems that oxidative stress plays a more complicated role than the one once believed and this role might be affected by the environment of each organism. Environmental factors such as UV radiation, air pollution, and an unbalanced diet, have also been implicated in the pathophysiology of aging and seem to initiate this process more rapidly and even at younger ages.

**Aim:**

The purpose of this review is to elucidate the role of oxidative stress in the physiology of aging and the effect of certain environmental factors in initiating and sustaining this process. Understanding the pathophysiology of aging will contribute to the development of strategies to postpone this phenomenon. In addition, recent studies investigating ways to alter the antioxidant defense mechanisms in order to prevent aging will be presented.

**Conclusions:**

Careful exposure to harmful environmental factors and the use of antioxidant supplements could potentially affect the biological processes driving aging and slow down the development of age-related diseases. Maybe a prolonged lifespan could not be achieved by this strategy alone, but a longer healthspan could also be a favorable target.

## Introduction

Today, life expectancy for a generally healthy person has increased to 80 years, but this is mainly due to scientific advances in pharmaceutics and better living conditions, which improve the quality of life (Brown, 2015). According to a recent study, the aging population (over 65 years old) worldwide reached 576 million in 2010, while it is estimated that in 2050 this number will increase to 1.5 billion (United Nations, 2019).

The aging process is a dynamic, time-dependent process characterized by the gradual, ever-increasing cell damage, the progressive reduction of cell functions, and the increased susceptibility to morbidity (Valavanidis et al., 2012). Although aging is a relatively well-preserved process among all organisms, the underlying molecular mechanisms differ between species and are still an active field of investigation (Xu and Tahara, 2013; Deschênes and Chabot, 2017; Magalhães et al., 2018; Jazbec et al., 2019; Warraich et al., 2020). Both genetic and environmental factors seem to variably contribute to the aging process, with environmental factors being major contributors (Kirkwood, 2005a).

The aging process is characterized by changes that occur at the molecular level and include genomic instability, telomere damage, epigenetic modifications, and loss of proteostasis, among others (López-Otín et al., 2013). “Cellular (replicative) senescence” is a term originally used to describe the irreversible loss of the proliferative capacity of cells, which cease dividing after serial passaging in culture, and was firstly proposed by Hayflick (1965) back in the 1960s. Senescent cells undergo alterations, including secretome changes, and have been proposed to accumulate in aging tissues playing a key role in the initiation and progression of aging (Jeyapalan et al., 2007; Wang et al., 2009). Moreover, senescence-inducing stimuli have been shown to increase with age (Baker et al., 2004; Faggioli et al., 2012), and this further strengthens the causative relationship between these two.

The theory that oxidative stress is a key component of the phenomenon of aging was introduced in 1956 by the American scientist Denham Harman (Harman, 1956) and further refined in 1969 (McCord and Fridovich, 1969) with the discovery of the enzyme superoxide dismutase (SOD), which is involved in the *in vivo* conversion of superoxide anion radical (O_2_^●−^) to oxygen (O_2_), and is one of the main cellular antioxidant mechanisms. It has been suggested that reactive oxygen species (ROS) formed endogenously as by-products of normal metabolism, affect the process of aging due to the increased oxidative damage to biological molecules and the promotion of cellular senescence. The researchers observed that species with higher metabolic rates have lower life expectancy and age faster, as it was suggested that energy consumption itself was responsible for aging through the production of O_2_^●−^and other ROS (“rate of living” hypothesis): a faster respiration rate, associated with greater production of ROS, contributes to faster aging (Sohal, 1976).

Since then, several major modifications have been introduced to better explain the link between oxidative stress and aging, while other contributing factors have also emerged, such as telomere shortening (Bodnar et al., 1998; Jiang et al., 2008), loss of proteostasis (Tawo et al., 2017), and differential expression of miRNAs (Dhahbi et al., 2011) among others, which continuously shed more light on the underlying mechanisms of aging. Nevertheless, there remains a controversy over the exact role of oxidative stress in the aging process, since oxidative stress alone cannot fully explain why some people age faster than others and antioxidant therapies have not successfully decelerate aging in all animal models. Therefore, the main purpose of this literature review is to elucidate the role of oxidative stress in the pathophysiology of aging and how environmental factors such as UV radiation, air pollution, and diet initiate and sustain this process implicating ROS. A thorough presentation of ROS production and oxidative stress will precede, in order to enhance the understanding of the association between oxidative stress and aging. Deciphering the pathophysiology of aging will help to develop strategies in order to slow down this phenomenon. Moreover, results of recent studies aiming to develop antioxidant defense mechanisms or other interventions in order to prevent/decelerate aging will also be presented.

### Free Radicals and ROS: Production and the Role of Mitochondria

Free radicals are molecules that are naturally produced in organisms, and in low concentrations are essential for cellular functions and defense systems (Phaniendra et al., 2015). A free radical is defined as an atom or molecule with one or more unpaired electrons in its valence shell. They are highly reactive because they show a strong tendency to pair their unpaired electron(s) by interacting with other radical or non-radical molecules (Littler, 1978; Pham-Huy et al., 2008).

The chemical production of free radicals mainly occurs *via* two mechanisms. The first mechanism involves redox reactions, i.e., the gain of an electron from a molecule (reduction) or the loss of an electron from a molecule (oxidation), while the second mechanism involves the homolytic cleavage of a covalent bond, in such a way that each fragment gets one of the shared electrons of the bond. In biology, electron-gaining and electron-donating agents are called prooxidants and antioxidants, respectively (Pham-Huy et al., 2008; Simioni et al., 2018).

The vast majority of endogenous prooxidants contain oxygen and are called reactive oxygen species (ROS), while other prooxidants contain nitrogen (reactive nitrogen species, RNS) or even halogens such as chlorine (reactive chlorine species, RCS).

Reactive prooxidant species can be classified into two categories: (a) radical; and (b) non-radical species, according to the existence of unpaired valence electrons in their molecules. Radical species include molecules such as the superoxide anion radical (O_2_^●−^), hydroxyl radical (OH^●−^), peroxy radical (RO_2_^●−^), nitric oxide (NO^●−^), nitrogen dioxide (NO_2_^●−^), and nitrous oxide (N_2_O), as well as the hypochlorite anion (ClO^−^). The most common non-radical prooxidant species are ozone (O_3_), hydrogen peroxide (H_2_O_2_), hypochlorous acid (HOCl), peroxynitrite anion (ONOO^−^), and aldehydes (Simioni et al., 2018; Möller et al., 2019).

Endogenous production of ROS is mainly linked to cellular metabolism. During cellular respiration, O_2_ is used by mitochondria to produce the necessary energy in the form of adenosine triphosphate (ATP), which is intertwined with the formation of ROS, especially within the mitochondrial electron transport chain (part of oxidative phosphorylation; Hirst et al., 2008; Warraich et al., 2020).

Mitochondria are dynamic cellular organelles surrounded by a double membrane and located in the cytoplasm of eukaryotic cells. They are often referred to as “power plants”, as their primary function is to supply the cell with energy through oxidative phosphorylation and the formation of ATP. Human mitochondrial DNA (mtDNA) consists of 16,569 base pairs. There are many copies of mtDNA within the mitochondria of mammal cells (100–10,000 copies), which are packaged with protein into spheroid bodies called nucleoids. There are 13 proteins, 22 tRNAs, and two rRNAs encoded by human mtDNA and are essential for the structural and functional maintenance of mitochondria (DiMauro and Schon, 2003; Kaufman et al., 2007; Chinnery and Hudson, 2013; Srivastava, 2017). Various other metabolic pathways apart from oxidative phosphorylation also occur in the mitochondria, including β-oxidation of fatty acids, and the tricarboxylic acid cycle (Nsiah-Sefaa and McKenzie, 2016; Martínez-Reyes and Chandel, 2020).

**Figure 1:**
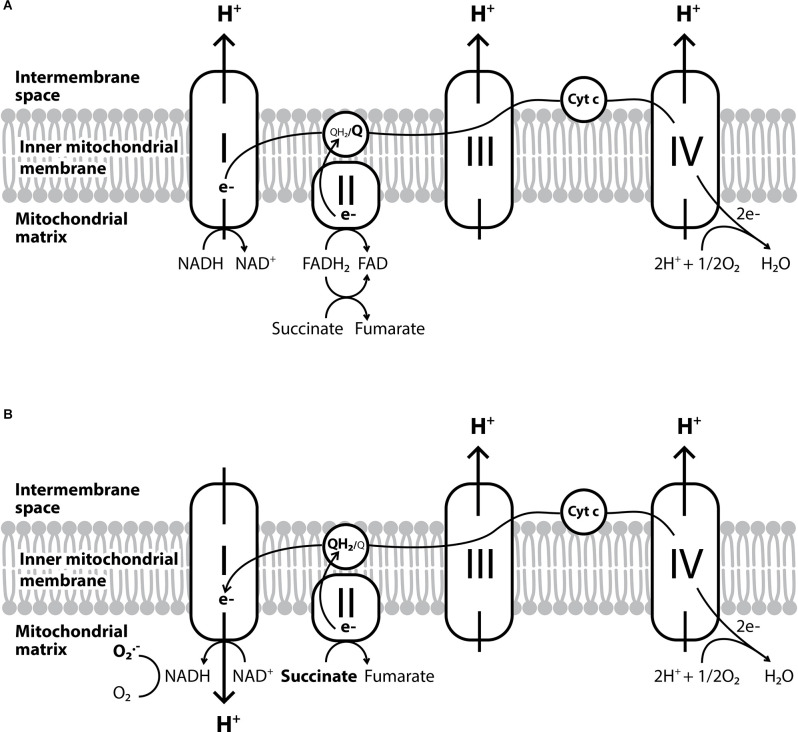
The electron transport chain and the reverse electron transport. **(A)** The conventional forward electron transport by the electron transport chain. **(B)** The reverse electron transport which leads to the overproduction of O_2_^●−^ and occurs when coenzyme Q gets over-reduced by complex II or the succinate is overused due to hypoxia.

The electron transport chain is located in the inner mitochondrial membrane. As the reduced nicotinamide adenine dinucleotide (NADH) and flavin adenine dinucleotide (FADH_2_) are oxidized, electrons flow sequentially into the respiratory chain complexes. In the last step, in complex IV (cytochrome c) electrons are transferred to molecular O_2_ leading to the production of H_2_O (Figure 1A). However, before electrons reach complex IV, there can be a “leakage” of O_2_ in complexes I and III, leading to the formation of O_2_^●−^ instead of H_2_O (Chance et al., 1979; Rinnerthaler et al., 2015). The O_2_^●−^ formed in the membrane is rapidly converted by SOD, SOD2 in the mitochondrial matrix and SOD1 in the transmembrane space, to H_2_O_2_. Hydrogen peroxide produced by SOD can react further to form HO^●^ both in the mitochondria and in the cytoplasm (Zsurka et al., 2018).

Another mitochondria-specific ROS production process is the reverse electron transport (Figure 1B). It is known so far, that this process mainly occurs when coenzyme Q gets over-reduced by complex II (Chouchani et al., 2014). Other enzymes implicated are glycerol-3-phosphate dehydrogenase, electron-transferring flavoprotein, or dihydroorotate dehydrogenase, which along with the inhibition of complexes III or IV can also induce reverse electron transport and further increase ROS production within mitochondria (Taylor and Moncada, 2010). Especially under hypoxic conditions, the metabolic shift of cells leads to the overuse of succinate, which can alter ROS production from complex I in the mitochondria to the reverse direction (Fernández-Agüera et al., 2015).

**Figure 2:**
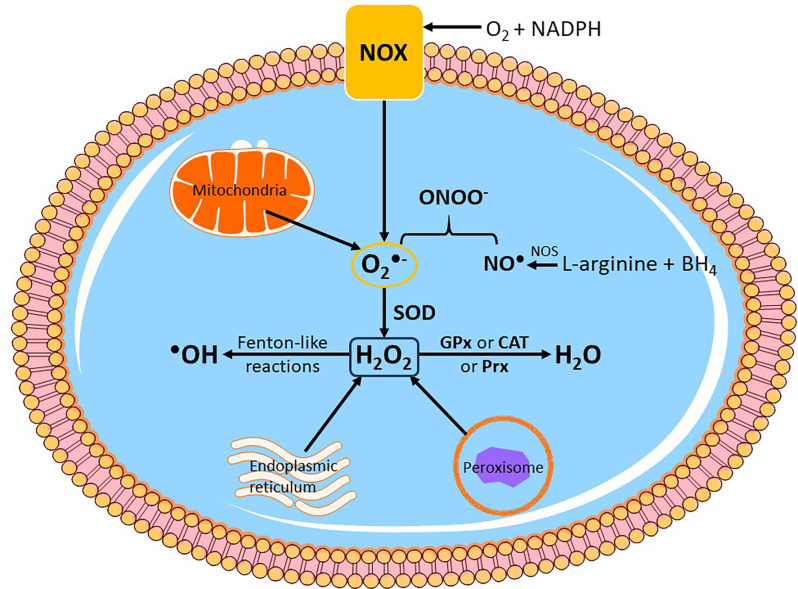
Endogenoussources of reactive oxygen species (ROS) production. Intracellular ROS is mainly produced by subcellular organelles such as the mitochondria, endoplasmic reticulum and peroxisomes. Another enzymatic system that produces ROS and is located on the cytoplasmic membrane is NADPH oxidase (NOX), which primarily generates superoxide anion radical (O_2_^●−^). In mitochondria, O_2_^●−^ is mainly produced by complexes I and III. Nitric oxide synthase (NOS) catalyzes the formation of nitric oxide (NO^●^) from L-arginine and tetrahydrobiopterin (BH_4_), and subsequently NO^●^ can yield peroxynitrite (ONOO^−^) by direct reaction with O_2_^●−^. Cytosolic O_2_^●−^ is then converted to hydrogen peroxide (H_2_O_2_) by endogenous superoxide dismutase (SOD). H_2_O_2_ can be further reduced to water (H_2_O) by the antioxidant enzymes glutathione peroxidase (GPx), catalase (CAT) or peroxiredoxin (Prx), or react with metal cations in Fenton and Fenton-like reactions to generate hydroxyl radical (^●^OH), which can cause immediate oxidative damage to biomolecules.

In addition to the mitochondrial respiratory chain and reverse electron transport, NADPH oxidase (nicotinamide adenine dinucleotide phosphate oxidase) is another important source of intracellular ROS. NADPH oxidases, also known as NOX enzymes, act by catalyzing the transfer of electrons from NADPH to molecular O_2_ to generate O_2_^●−^ and other ROS. NOX enzymes are located in the cytoplasmic membrane, endoplasmic reticulum, and mitochondria (Nauseef, 2008; Rinnerthaler et al., 2015). Moreover, other mitochondrial agents, such as monoamine oxidase or α-ketoglutarate dehydrogenase complex, contribute significantly to the total production of ROS in a way that depends on the tissues they are found (Meo and Venditti, 2001; Murphy, 2009; Zorov et al., 2014). Therefore, ROS production is interchangeably linked to mitochondrial function and mitochondria are the main source of intracellular ROS and even contribute to 90% of all ROS produced within the cell (Birch-Machin and Swalwell, 2010; Naidoo and Birch-Machin, 2017; Srivastava, 2017). Figure 2 summarizes the main pathways of intracellular ROS production and interconversion.

Oxygen and its compounds are thus, essential for aerobic organisms in order to maintain the numerous functions of cells, tissues, and organs through cellular respiration. Moreover, ROS have other physiological roles, i.e., they are important signaling molecules (Lee et al., 2002; Kim et al., 2008; Reczek and Chandel, 2015) that contribute to a variety of functions such as protein phosphorylation and activation or deactivation of signaling pathways (Banan et al., 2001; Takada et al., 2003; Bleier et al., 2015). For example, the production of ROS *via* the reverse electron transport at complex I provokes the differentiation of myoblasts (Lee et al., 2011), while sensing of oxygen levels by the carotid body is again regulated by these ROS (Fernández-Agüera et al., 2015). However, in addition to their concentration, the site of ROS production is also significant for their physiological roles (Scialò et al., 2016, 2017).

**Figure 3:**
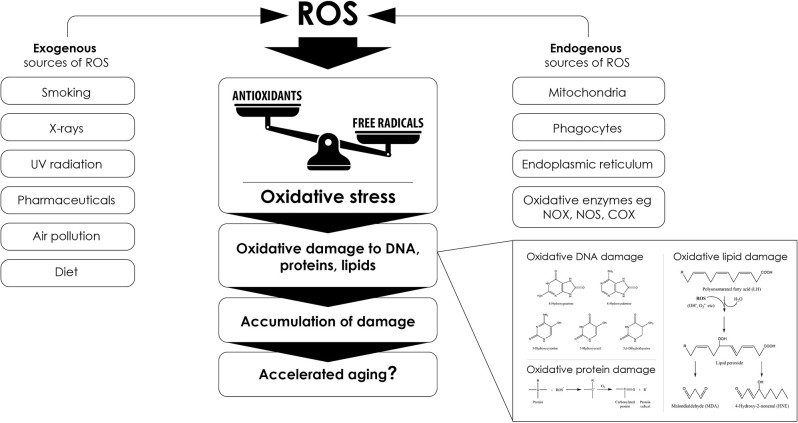
Sourcesof reactive oxygen species (ROS) that lead to oxidative stress and promote aging. Several endogenous and exogenous factors contribute to excess ROS formation. Any imbalance between the production of free radicals and ROS and their elimination by antioxidant systems can cause oxidative stress that ultimately promotes accelerated aging phenotypes through oxidative macromolecular alterations, such as the exemplary alterations depicted in the bottom right part of the figure.

Endogenous ROS can be also produced during intense physical activity (Meo and Venditti, 2001; Parker et al., 2014; Simioni et al., 2018) and exposure to phagocytic-activating microbes (Kraaij et al., 2010), but there are also a number of external factors that can lead to free radical and ROS production within the body. Such exogenous sources of free radicals are cigarette smoke, X-rays, UV radiation, various chemicals and pharmaceuticals, as well as particles of air pollution (ozone, nitroxides; Figure 3; Phaniendra et al., 2015).

### Antioxidant Mechanisms

Apart from their physiological roles though, under certain conditions, ROS can also become toxic (Fridovich, 1998). Hence, for proper biological function, it is necessary to maintain a balance between the formation and elimination of ROS (redox balance). It is estimated that about 10,000 free radicals “bombard” each cell per day (Poon et al., 2004), so various antioxidant defense mechanisms have been developed to maintain this redox balance (Pham-Huy et al., 2008). An antioxidant is defined as any substance that is present in small concentrations compared to the substrate that is oxidized, and that significantly delays or prevents the oxidation of this substrate (Brainina et al., 2019). Antioxidants work either by blocking the formation of ROS or by stopping the propagation of free radicals formed by radical chain reactions. Antioxidants can be classified according to their origin and chemical composition. Thus, there are endogenous and exogenous antioxidants, which we absorb with nutrition (Kurutas, 2016).

Antioxidants include enzymatic and non-enzymatic molecules. The most important antioxidant enzymes such as SOD, glutathione peroxidase (GPx), catalase (CAT), and various peroxiredoxins, protect intracellular mechanisms and maintain cellular redox balance (Valavanidis et al., 2012). SOD catalyzes the dismutation of O_2_^●−^ into O_2_ and H_2_O_2_. CAT then converts H_2_O_2_ to H_2_O and O_2_, a reaction that occurs in just a few seconds (Chelikani et al., 2004; Simioni et al., 2018). Many studies have shown that SOD plays an important role in protecting cells from oxidative stress during the development of the aging process, as will be discussed below (Rizvi and Maurya, 2007; Das and Muniyappa, 2013).

**Figure 4:**
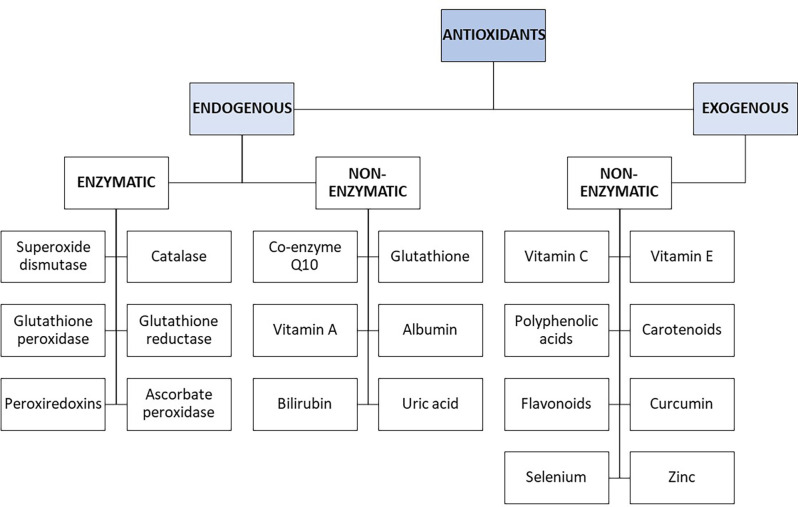
Schematic diagram of endogenous and exogenous enzymatic and non-enzymatic antioxidants.

Moreover, intracellular low molecular weight non-enzymatic antioxidants, such as ascorbic acid (vitamin C), uric acid, flavonoids, carotenoids, glutathione (GSH), and pyruvate, are particularly effective against oxidizing agents (Valavanidis et al., 2012; Kurutas, 2016). The main dietary antioxidants are fat-soluble or water-soluble herbal compounds such as tocopherols (vitamin E), β-carotene (precursor of vitamin A), lycopene, vitamin C, lutein, and various polyphenols (flavonoids and other related compounds; Simioni et al., 2018; the main endogenous and exogenous antioxidants are summarized in Figure 4).

Particular mention should be made to the plasma membrane redox system (PMRS), an antioxidant system on the plasma membrane. Analogous to the electron transport chain in the inner mitochondrial membrane, PMRS comprises redox enzymes involved in electron transport from intracellular reducing agents to extracellular acceptors, and energy metabolism. It is also important for the recycling of antioxidants such as coenzyme Q and α-tocopherol (vitamin E), which protects the plasma membrane from oxidative damages such as lipid peroxidation. Cells neutralize extracellular ROS through PMRS by transferring electrons from NAD(P)H and vitamin C to extracellular free radicals and ROS. Thus, PMRS acts as a protective antioxidant system for the cell, especially when mitochondrial dysfunction is present and cellular energy metabolism is compromised (de Grey, 2005; Hyun B.-H. et al., 2006; Hyun D.-H. et al., 2006).

### Oxidative Stress and Oxidative Damage

The redox balance is a key characteristic of cellular homeostasis and should be carefully maintained in order to ensure optimal cellular function. Reducing ROS levels below the normal threshold for homeostasis can disrupt the signaling role of oxidizing molecules. Similarly, an increase in ROS above indicated levels can prove harmful and lead to cellular death or a more rapid onset of certain related diseases.

The term “oxidative damage” or “oxidative stress” refers to the accumulation of free radicals and ROS in the body, due to an imbalance between the levels of these oxidizing agents and antioxidant mechanisms (McCord, 2000). It was introduced in 1970 to describe many harmful processes. However, it was later better clarified as the mechanism that occurs due to either the increased production of free radicals or the reduced ability of antioxidant mechanisms to eliminate them. In addition, oxidative stress can be a result of increased production of free radicals with a generally normal antioxidant function, or of the normal production of free radicals with comparatively low antioxidant capacity (Czerska et al., 2015; Weidinger and Kozlov, 2015).

**Figure 5:**
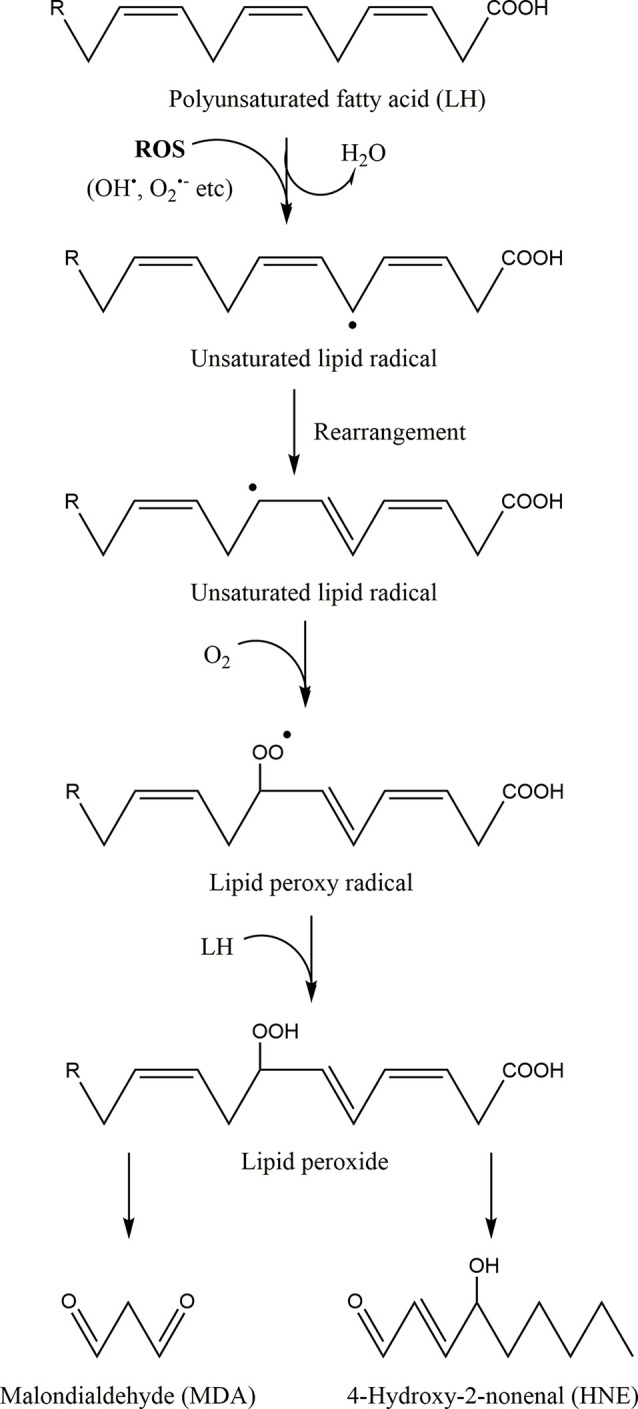
The process of lipid peroxidation of polyunsaturated fatty acids. First a free radical or reactive oxygen species (ROS) remove an electron from a methylene group between two double bonds of a polyunsaturated fatty acid (LH) leading to the formation of a new free radical (unsaturated lipid radical). The lipid radical formed is very unstable, resulting in its rapid rearrangement and the formation of a similar unsaturated lipid radical. This radical reacts with molecular oxygen towards a lipid peroxy radical. This peroxy radical can extract a hydrogen atom from an adjacent polyunsaturated fatty acid, thereby creating a new free radical and a lipid peroxide. These radical chain reactions further propagate and their main degradation end-products are malondialdehyde (MDA) and 4-hydroxy-2-nonenal (HNE), which are relatively stable. Hence, MDA and HNE can be used as a means of quantifying the levels of oxidative stress and lipid peroxidation within cells.

Oxidative stress poses a risk to cellular homeostasis, as it can cause random damage to key biomolecules by altering or inactivating them (Figure 3; Valko et al., 2007; Simioni et al., 2018). For example, it has been demonstrated that ONOO^−^, the product of the reaction between Ο_2_^●−^ and NO, induces tyrosine nitration and phosphorylation in proteins from synaptosomes leading to neuronal degradation (di Stasi et al., 1999). Similarly, DNA bases are also susceptible to oxidative damage, as guanine for example rapidly interacts with ONOO^−^ to form 8-nitroguanine, which can consequently be used as a biomarker for oxidative DNA damage (Yermilov et al., 1995). In particular, mtDNA is more sensitive to oxidative stress and 10 times more prone to mutations compared to nuclear DNA (Goo et al., 2013; Latimer et al., 2015). This is mainly due to the fact that: (a) it is not protected from the solid organization and wrapping around histones, as is the case for nuclear DNA (Taylor and Turnbull, 2005), although the mitochondrial transcription factor A (Tfam) has been shown to play a similar role to histones for mtDNA (Kaufman et al., 2007) and even sometimes protects it from oxidative damage (Hayashi et al., 2008; Xu et al., 2009); (b) it is very close to the inner mitochondrial membrane, one of the main sites of ROS formation (Markevich et al., 2020); and (c) mitochondria have less effective DNA repair mechanisms, for example, they can repair single-strand breaks but not double-strand breaks (Bohr, 2002; Sykora et al., 2012). Finally, ROS can also oxidize polyunsaturated fatty acids triggering lipid peroxidation, a chain of oxidative degradation of lipids, affecting cell membrane permeability (Figure 5; Simioni et al., 2018; Yalcinkaya et al., 2019; Juan et al., 2021). Such ROS-induced oxidative damage can eventually lead to cell death (Ding et al., 2016; Redza-Dutordoir and Averill-Bates, 2016; Zhao et al., 2016).

Based on the above damages, oxidative stress has been associated with numerous degenerative and chronic disorders, including autoimmune disorders (Ramani et al., 2020), chronic inflammatory conditions (García-Sánchez et al., 2020), cancer (Aboelella et al., 2021), arthritis (Fonseca et al., 2019), neurodegenerative (Wang X. et al., 2020) and, and cardiovascular diseases (de Almeida et al., 2020). For instance, increased production of ROS may enhance the synthesis of inflammatory cytokines (Chandel et al., 2000; Ichimura et al., 2003; Wang et al., 2010). Inflammation, in turn, can accelerate the formation of ROS, as proinflammatory cytokines such as TNF-α (tumor necrosis factor α) and IFN-γ (interferon γ) have been shown to increase ROS production by mitochondria and NOX (Yang et al., 2007; Mittal et al., 2014), establishing a link between oxidative stress and inflammation.

Additionally, oxidative stress-induced errors in mitochondrial gene expression can lead to malfunctioning mitochondrial subunits, and since mtDNA integrity is required for proper mitochondrial function, several diseases have been linked to mtDNA mutations (Bandy and Davison, 1990; Taylor and Turnbull, 2005; Chapman et al., 2020). Mitochondria divide separately from cellular division, so if damaged mitochondria proliferate faster than intact normal ones, it is possible that an increase in the rate of cell loss will occur due to their accumulation within the cell (Ma et al., 2020). mtDNA mutations create a vicious cycle in which malfunctioning mitochondria contribute to the generation of ROS, which leads to more ROS-mediated oxidative damage to the mitochondria (Birch-Machin and Swalwell, 2010; Kandola et al., 2015). Furthermore, alterations in coenzyme Q synthesis can alter the mitochondrial production of ROS, especially *via* the reverse electron transport, and is thus, associated with mitochondrial diseases (Fernández-Ayala et al., 2014). Mitochondrial dysfunction has been specifically linked to the aging phenotype and its more rapid evolvement (Kujoth et al., 2005; Hiona et al., 2010; Lane et al., 2015; Payne and Chinnery, 2015; Sun et al., 2016), and the pathogenesis of age-related diseases, such as Alzheimer’s disease (Onyango et al., 2016). Many studies have indeed shown that ATP production is affected by mutations in mtDNA, which lead to premature aging and neurodegeneration (Linnane et al., 1989; Münscher et al., 1993; Srivastava, 2017). However, PMRS is a major mechanism that can compensate for mitochondrial dysfunction as an alternative generator of ATP and maintain redox homeostasis by lowering oxidative stress in oxidative phosphorylation-deficient cells (de Grey, 2005; Hyun B.-H. et al., 2006; Hyun D.-H. et al., 2006).

As such, the theory that ROS formed within mitochondria contributes to the aging process has been developed. This theory has been modified, extended, and challenged by many researchers, but two basic principles remain generally accepted. First, oxidatively damaged damaged macromolecules accumulate during the aging process due to an imbalance between prooxidant and antioxidant agents, and second, the degenerative phenotype of aging is linked to this accumulated oxidative damage (Srivastava, 2017).

## Pathophysiological Mechanisms That Lead to Aging

More than 300 theories, either mechanistic or evolutionary, have been proposed by the scientific community to explain how and why living organisms age (Tosato et al., 2007). However, no such theory is universally accepted. The prevailing theories for the interpretation of the physiological phenomenon of aging of multicellular organisms and humans fall into two broad categories: (a) program-based theories; and (b) damage-based theories. Program-based theories claim that aging is the result of a genetically programmed process (cellular senescence), which is a normal mechanism in order to halt cell proliferation as a response to errors occurring during replication, neuroendocrine and immunological changes. Damage-based theories suggest that aging is due to the accumulation of damages to key biomolecules such as proteins, enzymes, membrane lipids, nuclear, and mtDNA, which cannot be repaired and result in dysregulated metabolism and contribute to cell degeneration and death (Kirkwood and Austad, 2000; Kirkwood, 2005b; Longo et al., 2005; reviewed in Valavanidis et al., 2015). Genetic inheritance seems to contribute only 3% to aging, whereas epigenetic and post-translational modifications are more important factors that contribute to aging (Wood et al., 2015; Pal and Tyler, 2016).

As an example of damage-based theories, p66Shc is a pro-apoptotic protein that is involved in the production of ROS in mitochondria, leading to mitochondrial damage and apoptosis under oxidative stress conditions (Galimov, 2010). As explained above, aging could be associated with a dysregulated mitochondrial function, for example when reduced oxidative phosphorylation occurs, which results in increased ROS production. Elevated ROS levels can activate p66Shc, thereby inducing ROS production even further, leading to apoptosis and maintaining a steady aging process. Thus, the p66shc protein appears to be an important link between ROS and aging (Ray et al., 2012). In addition, oxidative damage has been found to lead to telomere damage and shortening, which contributes to the aging process, and the development of age-related diseases (von Zglinicki, 2002; Cattan et al., 2008; Jiang et al., 2008; Ludlow et al., 2014; Blackburn et al., 2015; Pineda-Pampliega et al., 2020).

However, it seems that the relationship between ROS production and aging is non-linear, and various levels of oxidative stress might have opposing effects. Low levels have been proven to protect cellular structures by triggering defense systems, whereas higher concentrations can cause oxidative damage accelerating the aging phenotypes (Yun and Finkel, 2014). So, even though the exact underlying mechanisms of cellular aging remain to be explored, research showed that not only oxidative stress, but also mitochondrial dysfunction, DNA damage, oncogene expression, and loss of tumor suppressor genes such as PTEN (phosphatase and tensin homolog), RB1 (gene of retinoblastoma protein), NF1 (neurofibromin 1 gene), and the type I inositol-3,4-bisphosphate 4-phosphatase (INPP4A) gene, can induce cellular aging (Davalli et al., 2016). On the other hand, mitochondrial dysfunction associated with mtDNA mutations has been shown to induce aging phenotypes also independent of ROS production (Trifunovic et al., 2005).

Therefore, the theory of oxidative stress, as initially proposed to explain the aging process, was compromised in 2014 (Gladyshev, 2014). This was because a series of studies have shown that the involvement of ROS in the aging process depends on cellular metabolism, genotype, and function of defense mechanisms. For example, in many works, the overexpression of antioxidant enzymes or antioxidant supplementation did not affect aging or increase lifespan. In several model organisms, such as *Saccharomyces cerevisiae* (Koc et al., 2004), *Drosophila melanogaster* (Mockett et al., 1999, 2003) or *Caenorhabditis elegans* (Keaney and Gems, 2003; Keaney et al., 2004), growing under completely anaerobic conditions, antioxidant enzyme overexpression, supplementation with SOD mimetics, or antioxidant supplementation did not result in an increased lifespan. On the contrary, increased ROS production through respiratory complex I reverse electron transport was shown to extend *D. melanogaster* lifespan in another study (Scialò et al., 2016). However, more recent data still underline a causative relationship between the two (Deepashree et al., 2019).

Similarly, in vertebrates, there are a lot of gene manipulated models where the knockout of antioxidant enzymes did not result in a reduced lifespan. For example, CAT^−/−^ knockout mice (Ho et al., 2004), GPx1^−/−^ knockout mice (Ho et al., 1997), GPx2^−/−^ knockout mice (Esworthy et al., 2001), or SOD3^−/−^ knockout mice (Sentman et al., 2006) were not reported to live shorter lives, similar to CAT^+/+^ overexpressing mice which were not found to live longer (Chen et al., 2004).

Thus, it looks like oxidative stress is not simply linearly related to aging, and ROS, as by-products of cellular metabolism, can simply cause cell damage that may drive aging phenotypes (Carocho et al., 2018). Today, a slightly modified theory prevails, that the damage caused by oxidation is associated with aging and fragility (frailty) of the body (Viña, 2019). Frailty is a clinical syndrome concerning the elderly and is due to the cumulative deterioration of multiple physiological systems. It is characterized by diminished energy levels, reduced resistance to daily or acute stressors and causes vulnerability with a variety of adverse outcomes (falls, confusion, disability, loss of autonomy) and increased mortality (Clegg et al., 2013). Hence, oxidative stress remains one of the leading causes of aging.

### Cellular Senescence and Aging

Cellular senescence represents a stable and long-term loss of the proliferative capacity of cells which, nevertheless, are still viable and express metabolic activity (de Cecco et al., 2013). Some of the main inducers of cellular senescence are DNA damage and telomere erosion (Bautista-Niño et al., 2016). Oxidative stress is highly associated with cellular senescence, because it drives DNA lesions, accelerates telomere shortening, and activates molecular pathways leading to growth arrest (Chen et al., 2001; Nogueira et al., 2008; Barascu et al., 2012; Benkafadar et al., 2019). The senescence growth arrest has been shown to follow the activation of several signaling pathways, namely the chronic activation of p53/p21 and p16^INK4a^/pRB signaling pathways (McConnell et al., 1998; Takahashi et al., 2006; Popov and Gil, 2010), a persistent DNA damage response (DDR) signaling, and the stress-responsive p38MAPK (phosphorylated p38 mitogen-activated protein kinase) and protein kinase C signaling pathways (Passos et al., 2010; Freund et al., 2011).

Cellular senescence is a key contributor to organism aging, since senescent cells are robustly accumulated in aging tissues, especially in aging skin (Ressler et al., 2006; Velarde et al., 2012). Similarly, in aging primates or rodents, senescent cells have been found significantly expressed (Herbig et al., 2006; Wang et al., 2009; Salminen et al., 2011; Yousefzadeh et al., 2020; Yu et al., 2020). In a recent study, the therapeutic targeting of senescent cells in order to promote their apoptosis helped to reverse tissue homeostasis in both fast aging and normal aged mice, clearly implicating senescence with aging (Baar et al., 2017). Despite that, cellular senescence can also occur as a programmed event in order to promote tissue remodeling during embryonic development, indicating its multilevel role in mammals (Muñoz-Espín et al., 2013; Davaapil et al., 2017).

### The Senescence-Associated Secretory Phenotype (SASP)

Some of the basic characteristics of these cells are increased size, upregulated enzyme activity of lysosomal β-galactosidase (SA-β-GAL), high levels of p16, p21, histone variant macroH2A, and p38MAPK, as well as the continuous development of the SASP phenotype (senescence-associated secretory phenotype). The SASP is a common characteristic of senescent cells that undergo senesce mainly due to genomic damage or epigenetic modifications, and differentiates them from other types of non-proliferating cells. This phenotype represents the secretion of soluble factors including interleukins (ILs), chemokines and growth factors, enzymes such as matrix metalloproteinases (MMPs), and insoluble proteins such as extracellular matrix (ECM) components. Nuclear factor-kappa B (NF-κB) also plays an important role in the occurrence of the SASP phenotype (Coppé et al., 2008; Birch and Passos, 2017; Song et al., 2020). These factors sustain an immunomodulatory and proinflammatory microenvironment (Coppé et al., 2008; Davalos et al., 2010; Freund et al., 2010). This phenotype, although highly conserved among senescent cells, can slightly vary among different cell types and according to the senescence-inducing stimuli. As such, stimuli including DNA damage, shortened telomeres, epigenetic modifications or oxidative stress, can provoke SASPs of different strengths and profiles of excreted factors (Acosta et al., 2008; Wajapeyee et al., 2008; Coppé et al., 2010; Novakova et al., 2010; Lesina et al., 2016).

Many SASP factors act as proinflammatory mediators either directly or indirectly (Davalos et al., 2010). For example, an early sign of senescent cells is the increased expression of IL-1α, which *via* the activation of NF-κB upregulates the expression of other proinflammatory interleukins, such as IL-6 and IL-8 (Bhaumik et al., 2009; Orjalo et al., 2009; Su et al., 2019). This inflammatory microenvironment can sustain the continuous growth arrest characterizing senescent cells (Kuilman et al., 2008; Madani et al., 2021).

The SASP is of great significance to the role of cellular senescence in aging and age-related diseases (Baker et al., 2011; Salminen et al., 2011). It has been shown to drive degenerative dysfunctions associated with increasing age and escalate aging tissue deterioration (Krishnamurthy et al., 2006), and additionally *via* a mechanism called paracrine senescence. In this mechanism, senescent cells promote healthy neighboring cells to senescence through the secretion of chemokines (Hubackova et al., 2012; Nelson et al., 2012; Acosta et al., 2013; Lagnado et al., 2021; Waters et al., 2021).

### Oxidative Stress and Aging

Oxidative stress, apart from its link with cellular senescence as discussed above, is also one of the direct mediators of SASP (Coppé et al., 2010; Passos et al., 2010; McCarthy et al., 2013), for example *via* the persistent DDR ROS can sustain (Rodier et al., 2009). Both telomere-dependent and telomere-independent DDR signaling were shown to drive ROS production and mitochondrial damage, which created a vicious cycle between ROS production, DNA damage, and continuous DDR signaling, which promoted SASP (Passos et al., 2010). Moreover, antioxidant supplementation and low oxygen tension prevented the SASP-associated IL-1α, IL-6, and IL-8 expression (McCarthy et al., 2013).

**Figure 6:**
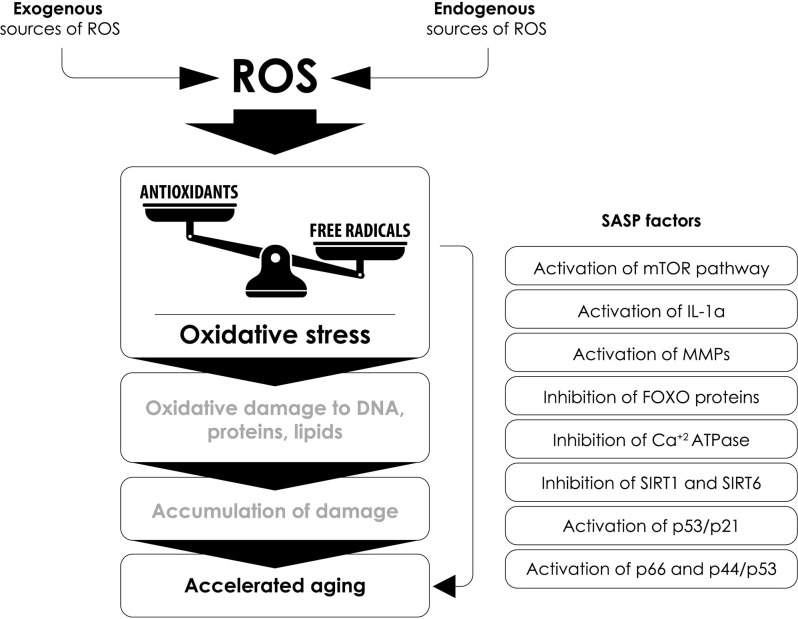
The interplay between oxidative stress, SASP factors and accelerated aging. Several senescence-associated secretory phenotype (SASP) factors that contribute to accelerated aging are affected by the increased production of reactive oxygen species (ROS) and oxidative stress. SASP, senescence-associated secretory phenotype; mTOR, mammalian target of rapamycin; IL-1α, interleukin-1α; MMPs, matrix metalloproteinases; FOXO, Forkhead box; Ca^++^-ATPase, calcium ATPase; SIRT1, sirtuin 1; SIRT6, sirtuin 6.

Based on the above, oxidative stress remains a key contributor to the pathophysiology of aging, and in more detail, this can be further underscored by the fact that increased levels of oxidative stress induce various factors that are associated with cellular aging, such as:

(a) the regulation of functions related to the mechanistic target of rapamycin (mTOR; Xu et al., 2014),
(b) the production of IL-1α, which leads to the formation of a pro-inflammatory environment by increasing the activity of NF-κB and the epithelial-mesenchymal transition (EMT; Laberge et al., 2015),
(c) the induction of the expression of MMPs (Dasgupta et al., 2010),
(d) the inhibition of FOXO (Forkhead box) proteins, which are involved in the protection against oxidative-stress-induced by the insulin/insulin-like growth factor (IGF-1) signaling pathway (Lin et al., 2018),
(e) the reduction of sarcoplasmic (SR) and endoplasmic reticulum (ER) activity and of the calcium-dependent ATPase (Ca^++^-ATPase) activity leading to cardiac aging (Babušíková et al., 2012),
(f) the inhibition of sirtuin activity (proteins with mono-ADP-ribosyltransferase or deacetylase activity involved in functions such as stress resistance, transcription, apoptosis, and inflammation; Guarente et al., 2018), that leads to: (1) increased ROS formation by inhibiting SOD (Qiu et al., 2010); (2) a pro-inflammatory state by preventing the inhibition of TNF-α and NF-κB (Yang et al., 2012); and (3) an oncogenic effect by preventing sirtuin’s inhibitory effect on oncogenes c-Jun and c-Myc (Min et al., 2012; Lin et al., 2013),
(g) the regulation of the p16^INK4a^/pRB and p53/p21 pathways leading to aging (Chen et al., 2006; Figure 6).

#### The Role of mTOR in Aging

One of the signaling pathways clearly linked to aging is the mTOR pathway, the activation of which is associated with overactivated cellular functions such as fibroblast hypersecretion, that alters homeostasis leading to age-related diseases and death (Leontieva et al., 2012). The activity of mTOR is more elevated in male mice than in females, due to increased levels of phosphorylated S6 (pS6) and phosphorylated AKT (phosphorylated protein kinase B, pAKT, in Ser473) in the tissues examined (heart and liver), which are indicators of the mTOR activity (Leontieva et al., 2012).

TOR kinase forms two complexes, mTORC1 and mTORC2 (Bhaskar and Hay, 2007). mTORC1 is sensitive to rapamycin and is a “sensor” of energy levels and the oxidative state of the cell that controls protein synthesis and cell growth (Jacinto et al., 2004). The activity of mTORC1 is critical for cell growth and proliferation, while dysfunction in the mTORC1 signaling pathway is associated with metabolic diseases, cancer, and aging (Bhaskar and Hay, 2007).

The interplay between mTOR and oxidative stress is complicated since in the lungs it was demonstrated that senescent lung epithelial cells had upregulated mitochondrial biogenesis driven by the mTOR pathway, which led to increased mitochondrial ROS production. As a consequence, mitochondrial-specific antioxidant therapy or reduced ROS-initiated molecular damage inhibited mTORC1 activation and cellular senescence in the lungs (Summer et al., 2019). Moreover, in cancer stem-like cells, ROS produced by NOX1 activated mTORC1 kinase (Ohata et al., 2019), similar to another study that reported the activation of mTORC1 by mitochondrial-generated ROS, which led to cellular senescence in human fibroblasts (Nacarelli et al., 2016). The study concluded that a way of delaying the onset of senescence is by decreasing mitochondrial ROS (Nacarelli et al., 2016). Older research though indicated that long-term or high ROS exposure decreased mTORC1 activity, whereas low doses of ROS could stimulate mTORC1 in several cells (Li et al., 2010). Therefore, the exact relationship between ROS and the mTOR pathway might be cell-type specific and definitely needs further investigation.

The circadian clock, the internal biological clock, has also been linked to the aging process, but the molecular mechanisms that regulate it remain mostly unknown (Khapre et al., 2014). BMAL1 molecule is a transcription factor comprising the regulatory unit of the circadian clock. Transgenic mice models lacking the BMAL1 gene have been associated with premature aging and reduced lifespan. A study using this transgenic model showed that there was increased activity of the mTORC1 complex, a finding that was associated with more rapid aging. In addition, administration of an inhibitor of mTORC1 increased lifespan by 50%. The findings of this study led to the conclusion that the circadian clock controls the activity of the mTOR pathway through mechanisms that depend on the factor BMAL1, and that this regulation is particularly important for the control of aging and metabolism (Khapre et al., 2014).

Several experimental studies have shown that the administration of mTOR inhibitors to mice extended life expectancy by 9%–14%, even when the administration started relatively late (Harrison et al., 2009; Anisimov et al., 2011; Miller et al., 2011; Johnson et al., 2013). In addition, the administration of an mTOR inhibitor has improved many age-related conditions in older animals, including tendon stiffness, heart failure, reduced cognitive function, and reduced mobility (Wilkinson et al., 2012; Johnson et al., 2013). For example, in a relevant study rapamycin, a known mTOR inhibitor, could improve age-related phenotypes, such as frailty, long-term memory, and tissue and muscle performance, in a genetically enhanced NF-κB mouse model. Although no extension in lifespan was recorded, rapamycin alleviated age-related phenotypes and improved mice healthspan independent of its activity as a suppressor of inflammation (Correia-Melo et al., 2019). Therefore, rapamycin is currently being investigated as an anti-aging drug (Selvarani et al., 2021; Zhang et al., 2021).

Another study in elderly volunteers investigated whether the administration of mTOR inhibitors had any beneficial effect on aging and age-related conditions. In particular, they used the inhibitor of mTOR RAD001, and tested whether there was any effect on the aging of the immune system of the elderly, assessing the response of volunteers to vaccination against the influenza virus. The inhibitor RAD001 improved the response of the elderly to the vaccine by 20% at doses that were relatively well-tolerated (0.5 mg daily or 5 mg weekly for 6 weeks). Furthermore, it decreased the percentage of CD4 and CD8 T-lymphocytes expressing the receptor of the programmed cell death protein-1 (PD-1), which inhibits T-cell signaling and is found to be increasing with age. Hence, administration of the mTOR inhibitor RAD001 could improve the immune response of the elderly (Mannick et al., 2014).

#### The Role of Inflammatory Response in Aging

A recent study elucidated the mechanism by which IL-1α, a cytokine that modulates immune responses, is activated during cellular aging. Mature IL-1α is produced after activation of the inflammasome and during cellular aging (Wiggins et al., 2019). The inflammasome is activated by increased ROS in a variety of cell types (Liang et al., 2017; Qiu et al., 2019; Xu T. et al., 2019). The researchers showed that IL-1α was activated by a cleavage catalyzed by the noncanonical inflammatory caspases 5 or 11 in a conserved region. Caspase 5 plays an important role in the release of IL-1α in human macrophages, while in mice macrophages caspase 11 appears to play this role. However, there is also a beneficial effect of this IL-1α signaling, since it was found necessary for the subsequent activation of macrophages for the removal of senescent cells (Wiggins et al., 2019).

The active contribution of mitochondria to the pro-inflammatory phenotype and cellular senescence was deciphered in another study, where it was found that the absence of mitochondria diminished several SASP factors, although the production of ATP was not severely altered due to enhanced glycolysis. In detail, the ROS-dependent AKT/mTOR pathway activation led to mitochondrial biogenesis, which contributed to the ROS-induced activation of DDR and the cell cycle arrest observed in senescent cells. As proof of concept, the reduction of cellular mitochondria prevented senescence in the liver of aged mice (Correia-Melo et al., 2016). Furthermore, even cell-free mtDNA was well correlated with the expression of pro-inflammatory cytokines *via* a number of inflammatory pathways (Pinti et al., 2014; White et al., 2014). Indeed, the interplay between mitochondria and the pro-inflammatory phenotype characterizing senescent cells seems to be more complicated than previously described (reviewed in Chapman et al., 2019).

Finally, even the telomere dysfunction characterizing senescent cells was also correlated with chronic, progressive low-grade inflammation, as this type of inflammation induced premature aging and cellular senescence in NF-κB subunit 1 knockout mice *via* a ROS-mediated telomere dysfunction pathway (Jurk et al., 2014).

The administration of rapamycin was able to alleviate the pro-inflammatory phenotype of aging cells. In particular, it reduced the secretion of inflammatory cytokines by aging cells. Rapamycin decreased IL-6 and the mRNA levels of other cytokines, but selectively suppressed the translation of membrane-bound IL-1α. Decreased IL-1α downregulated the transcriptional activity of NF-κB, which controls the SASP phenotype. Exogenous administration of IL-1α restored IL-6 secretion in rapamycin-treated cells. Moreover, rapamycin suppressed the ability of aging cell fibroblasts to induce the development of prostate cancer in mice. Based on the above, it was observed that the two pathways, involving mTOR and IL-1α, are interconnected (Laberge et al., 2015).

#### The Role of Matrix Metalloproteinases in Aging

MMPs and their endogenous inhibitors, the tissue inhibitors of metalloproteinases (TIMPs), regulate the deposition of structural proteins of the ECM (accumulation) and their degradation (turnover). The expression of MMPs and TIMPs, as well as their relative balance, seem to change during the aging process (Liu and Khalil, 2017).

Indeed, aging and various other pathologies are related to changes in the composition, structure, and mechanical integrity of the connective tissue. Collagen is ECM’s most abundant protein and contributes to the hardness and durability of tissues. Changes in the structure of collagen, as well as the susceptibility to degradation, are associated with age-related and chronic diseases such as cancer, Alzheimer’s disease, atherosclerosis, osteoarthritis, and emphysema. MMPs significantly contribute to tissue remodeling and collagen degradation (Cabral-Pacheco et al., 2020; Raeeszadeh-Sarmazdeh et al., 2020).

During aging and under the influence of oxidative stress, the accumulation of advanced glycosylation end products (which cause fibroblast apoptosis *via* ROS production and activation of the inflammasome; Dai et al., 2019) and the depletion of glycosaminoglycans caused the MMP-mediated degradation of collagen. Digestion with MMPs led to a disruption in the structural and mechanical integrity of collagen fibrils, which may exacerbate ECM pathology (Ruiz et al., 1999; Panwar et al., 2018).

#### Involvement of FOXO Proteins in Aging

FOXO factors regulate cell growth and proliferation and ultimately contribute to longevity (Davalli et al., 2016). Studies from the last two decades show that the conserved transcription factor DAF-16/FOXO is one of the main cellular components acting as stress sensors. It induces the activation of genes that promote resistance to stress, slow down cellular processes, and support longevity, resulting in better survival of organisms (Sun et al., 2017). The transcription factor DAF-16/FOXO is involved in the insulin/IGF-1 signaling pathway (ISS). Under normal conditions, ISS is active and leads to the phosphorylation of DAF-1/FOXO by AKT and SGK (serine/threonine-protein kinases), resulting in its binding with the 14-3-3 proteins in the cytoplasm, away from its target genes. However, under stress conditions such as mild oxidative stress, this transcription factor is released from the 14-3-3 proteins and enters the nucleus in order to regulate the expression of stress-resistance genes and other target genes that promote longevity (Mueller et al., 2014).

A recent study on *Caenorhabditis elegans* showed that the transcription factor HLH-30/TFEB, through complementary functions, acted synergistically with DAF-16/FOXO. The transcription factor TFEB is one of the major regulators of lysosome biogenesis and autophagy, which are important processes for metabolism and aging. The study showed that the transcription factors DAF-16/FOXO and HLH-30/TFEB formed a complex and worked together to regulate many target genes (Lin et al., 2018).

#### Changes in Ca^++^-ATPase Function Are Associated With Cardiac Aging

Heart aging is associated with many molecular, structural and ionic biophysical and biochemical changes, including the reduction in heart contractility. Phenomena such as left ventricular hypertrophy, atrial fibrillation, and heart failure are very common in aging (Dai et al., 2012). Several studies have shown an association between time-dependent damage to the rate of ventricular relaxation and a decrease in the ability of SR to bind calcium ions (Schmidt et al., 2000). The change in SR function is linked to reduced levels of SERCA 2a (sarco/endoplasmic reticulum Ca^++^-ATPase) or a decrease in the ratio of SERCA 2a to phospholamban. In addition, it may be associated with post-translational protein modifications (Slack et al., 2001). Proteins undergo several modifications under the influence of oxidative stress, as discussed previously. In particular, proteins with sulfur-containing amino acids (cysteine and methionine) and those containing aromatic amino acids, such as tyrosine, phenylalanine, and tryptophan, are more prone to oxidative damage. Aging causes an increase in reversible and irreversible oxidative modifications, such as the formation of protein carbonyls, and conversions of tyrosine to dityrosine or 3-nitrotyrosine (Sharov et al., 2006).

A study in rats showed that aging reduced Ca^++^-ATPase activity in the heart, a finding attributed to oxidative damage on proteins caused by oxidative stress, and not to changes in SERCA and phospholamban protein levels, since their expression and phosphorylation were similar in rats of 2, 6, 15, and 26 months of age (Babušíková et al., 2012).

#### The Role of Sirtuins in Aging

Sirtuins (SIRT1-SIRT7) are histone deacetylaces that regulate energy metabolism and mitochondrial function. They act as metabolic sensors, using intracellular metabolites such as NAD^+^ and acetyl-coenzyme A to regulate mitochondrial function depending on nutrient supply, so their activity depends on the cellular metabolic status. They also coordinate the stress response and damage repair mechanisms. Although there is currently no conclusive data that sirtuins can successfully decelerate human aging, there are certain studies that found a link between sirtuins and the aging process or neurodegenerative age-related diseases (Wang and Wei, 2020; Zhao et al., 2020). Thus, the activation of sirtuin proteins could be considered as a means to increase longevity (Grabowska et al., 2017).

Sirtuin 1 (SIRT1) has been found to regulate secretory proteins associated with SASP, silencing their genes in the promoter region, by provoking epigenetic modifications (Ventura et al., 2002; Chen et al., 2013). It also seemed to play an important role in modifying stress response by deacetylating p53, thus acting against aging and the onset of age-related diseases. High levels of ROS activate p53, which in turn activates p53-mediated apoptosis and cellular aging (Ota et al., 2006, 2007). Furthermore, SIRT1 has the ability to regulate ROS-dependent FOXO factors. ROS were able to reduce the activity of SIRT1, which resulted in cells acquiring an aging phenotype (Furukawa et al., 2007; Cencioni et al., 2013). Similarly, in mouse oocytes, the same relationship between the oxidative stress-protective activity of SIRT1 and its downregulation during aging was found (Di Emidio et al., 2014). SIRT1 was also found decreased in prematurely senescent ARPE-19 cells (retinal pigment endothelial cells-ECs), an activity orchestrated by miR-34α, which conferred increased susceptibility to oxidative stress (Tong et al., 2019).

Sirtuin 6 (SIRT6) is one of the few genes known to be involved in both longevity and progeria (Hutchinson-Gilford syndrome), a genetic disease that resembles accelerated aging (premature aging; Liao and Kennedy, 2016). In particular, overexpression of SIRT6 led to longevity, a finding attributed to low serum levels of IGF1, though this phenomenon occurred only in male mice and not in females (Kanfi et al., 2012). On the other hand, mice lacking the *SIRT6* gene showed characteristics of progeria in their phenotype (Mostoslavsky et al., 2006). Functionally, SIRT6 protein plays an important role in DNA repair, telomerase functionality, genome stability, and cellular aging (Tennen and Chua, 2011). More recent studies have shown that SIRT6 is associated with oxidative stress (Liao and Kennedy, 2016; Pan et al., 2016). In particular, SIRT6 deacetylase activity was associated with redox homeostasis/oxidative stress in human mesenchymal stem cells, suggesting that this activity may regulate longevity and progeria. Moreover, SIRT6 was able to act as a cofactor of Nrf-2, as it was found in a protein complex with nuclear factor Nrf-2 and RNA polymerase II, both necessary for the activation of genes with antioxidant activity such as heme oxygenase-1 (Pan et al., 2016).

The role of SIRTs in EC senescence was also explored in human retinal microvascular ECs (HRECs), where again it was shown that glucose-induced oxidative stress downregulated SIRTs (SIRT3, 4, 5) *in vitro* and *in vivo*, increased SIRT-targeting miRNAs and led to early HREC senescence, endothelial-to-mesenchymal transition, and oxidative damage contributing to aging (Liu et al., 2020). However, not all SIRTs share the same role, for example SIRT2 was shown to have opposite effects to SIRT1 in the stress response pathway, age-related diseases, and aging (Outeiro et al., 2007; Wang Y. et al., 2020; Kaitsuka et al., 2021).

#### The Role of P53 Family of Proteins in Aging

Oxidative stress is an important inducer of tumor suppressor protein p53, which is a nuclear phosphoprotein that determines cell cycle evolution. During the process of cellular aging and in the presence of DNA damage, the *p53* gene is activated and regulates the transition of the cell from phase G1 to phase S of the cell cycle (Gambino et al., 2013). Yet an alternative way of inducing cellular aging is through the activation of proto-oncogenes. A typical example is the proto-oncogene *p16^INK4a^*, which was considered essential for the progression of cellular aging, due to its ability to activate the tumor suppressor protein pRB (LaPak and Burd, 2014). Furthermore, the *Ras* proto-oncogene was also involved through the overexpression of Cdc6 and the inhibition of nucleotide metabolism (Aird et al., 2013). Therefore, stress is considered as an inducer of cellular aging and is also responsible for epigenetic modifications. More specifically, changes in chromatin levels due to exposure to histone deacetylase inhibitors led to cellular aging *via* p21, a cyclin-dependent kinase inhibitor which is one of the targets of p53 activity and associates DNA damage to cell cycle arrest (Waldman et al., 1995; Bunz et al., 1998; van Deursen, 2014).

Resistance to oxidative stress was associated with the slowing of aging in mammals and the absence of accelerated oncogenesis, indicating the inactivation of downstream targets of the p53 pathway. The regulation of p53 was studied in mice lacking the *p66* gene, a mutation that slowed aging and provided cellular and systemic resistance to oxidative stress (Gambino et al., 2013). A transcriptional network consisting of approximately 200 genes was identified, which are suppressed by p53 and encode critical factors for cell growth through mitosis or suppression of aging. These genes underwent selective downregulation, both *in vitro* in fibroblast cultures after oxidative stress stimulation, and *in vivo* in tissues during the normal aging process. This selectivity was due to the non-expression of p66 and the activation of p44/p53 (also called Delta40p53), an isoform of p53 that contributes to premature aging and prevents mitosis after protein damage. Lack of p66 slowed aging and increased p44/p53 transgenic mice longevity (Gambino et al., 2013).

TAp73 (a tumor suppressor protein derived by the *p73* gene locus) belongs to the p53 family, and has a protective effect against aging, regulating mitochondrial activity and preventing the accumulation of ROS (Budanov, 2014). Transgenic mice models lacking the *TAp73* gene had an increased oxidative load and showed an aging phenotype. TAp73 deficiency reduced cellular levels of ATP, oxygen consumption, and the activity of mitochondrial complex IV, which resulted in increased ROS production and sensitivity to oxidative stress. The Cox4i1 subunit of mitochondrial complex IV is a direct target of TAp73. In conclusion, TAp73 protein affected the mechanism of mitochondrial respiration and redox homeostasis, thus regulating the aging process (Rufini et al., 2012).

Recently, another signaling pathway has been described that involves miRNAs in the cellular process of aging. In particular, a study of the profile of miRNAs and mRNAs of aged mice revealed both their relative expression and the ability of miR-124 to induce cellular aging by targeting the Ccna2 protein. Activation of p53 has been shown to induce the expression of many miRNAs, including miR-124 and miR-29a/b/c, which target Ccna2, the antagonist of p21, resulting in cellular aging (Xu S. et al., 2019).

#### The Role of miRNAs in Aging

Further to the contribution of miRNAs in aging, miRNAs have been shown to share a role in the regulation of lifespan (Boehm and Slack, 2005; Ibáñez-Ventoso et al., 2006; Lehrbach et al., 2012), and that alterations in cellular miRNA expression can affect mammalian aging (Bates et al., 2010; Inukai et al., 2012; Smith-Vikos et al., 2016; Cosín-Tomás et al., 2018), and age-related diseases such as age-related macular degeneration (Mrowicka et al., 2021; Tisi et al., 2021). On the other hand, aging was also related to altered miRNA expression (Hackl et al., 2010). In the following studies, the link between oxidative stress, miRNA expression, and aging becomes even more profound.

Mesenchymal stem cells (MSC)-derived exosomes were found to significantly decrease aging-related CD_4_^+^ T cell senescence by reducing oxidative damage, SASP expression, aging-related proteins such as p53, and other aging markers. This activity was linked to miR-21, which downregulated PTEN and increased PI3K and AKT activation, thus leading to Nrf2-induced antioxidant gene expression (Xiong et al., 2021).

Similarly, MSC small extracellular vesicles were found to decrease biomarkers of senescence and SASP in oxidative stress-induced senescent ECs, while miR-146a was shown to be the mediator of this activity by downregulating Src activation and other downstream targets. *In vivo*, senescent ECs in natural aging and type-2 diabetes mouse wound-healing models were restored and this led to accelerated wound closure and rescued angiogenesis (Xiao et al., 2021).

Another study proving the link between miRNAs and aging observed that the restoration of cellular NAD^+^ levels, which are reduced during aging, can restore vessel functions in aged mice, thus rescuing age-related dysregulations. Treatment with nicotinamide mononucleotide (NMN), to increase cellular NAD^+^, restored several miRNAs, whose expression was dysregulated in aged mice, and these changes are expected to promote anti-atheromatic effects and vascular epigenetic rejuvenation, by exerting vasoprotective and antioxidant activity. Therefore, the restoration of aging-related NAD^+^ depletion helped to abrogate oxidative stress and save from age-related vascular dysfunctions through a miRNA-dependent mechanism (Kiss et al., 2019).

Oxidative stress, miRNAs, and aging were also found interconnected in human fibroblasts, where the silencing of DNA methyltransferase 2 (DNMT2) led to increased susceptibility to oxidative damage, inhibition of cell proliferation, and induced cellular senescence *via* altering miRNA expression. Several miRNAs were found upregulated, for example, miR-28-3p, miR-30b-5p, and miR-200c-3p, which are related to proliferation. DNMT2 was also found upregulated in senescent cells, meaning that the miRNA-related modifications induced by DNMT2 can determine longevity in human fibroblasts (Lewinska et al., 2018).

Additionally, the roles of miR-24 and miR-424 were elucidated in oxidative stress-induced premature senescent fibroblasts, where it was shown that miR-24 upregulated p53 and induced senescence, whereas miR-424 acted in the opposite manner. The downstream target of miR-24 was identified to be DNA topoisomerase 1, whose expression was induced by oxidative stress, and when downregulated due to miR-24, cellular senescence was accelerated (Bu et al., 2016).

Finally, the effect of ROS on miRNA expression in ECs revealed that the miR-200 family is upregulated under oxidative stress, which leads to EC growth arrest, apoptosis, and senescence. This activity was found related to ZEB1 downregulation by miR-200c, which involved p53 and retinoblastoma proteins *in vitro*. *In vivo*, again oxidative stress induced the expression of the miR-200 family leading to EC senescence, thus providing a causative relationship between oxidative stress, altered miRNA expression, and aging (Magenta et al., 2011).

### The Role of Environmental Factors in Oxidative Stress and Aging

Life expectancy is constantly increasing and today a person can live up to 120 years. Nevertheless, many environmental factors contribute to the initiation of aging (“exogenous aging”; Naidoo and Birch-Machin, 2017). This could be partly due to the fact that these factors are modifiable. Environmental factors that accelerate aging are those that lead to macromolecular damage or interfere with cellular repair mechanisms. They include chronic infections, certain heavy metals, UV radiation, and various other conditions that increase oxidative stress, such as smoking (Karol, 2009). Thus, if indeed the phenomenon of aging is affected by oxidative stress, then environmental, pharmacological, and nutritional strategies could possibly slow down the phenomenon (Maurya et al., 2016). According to Harman’s theory, scientists believed that by removing harmful oxidative molecules from the body, they could mitigate cell damage and slow down the aging process. However, this theory applies only to the skin aging process, according to the data we have so far. We know that skin has a higher oxidative load than other tissues and organs, and that environmental factors such as exposure to UV radiation contribute up to 80% to skin aging. Therefore, by reducing exposure to UV radiation we can slow down the skin aging process (Amaro-Ortiz et al., 2014; Gu et al., 2020).

#### Heavy Metals

Heavy metals have a prooxidant effect and have been found to exacerbate age-related oxidative stress (Karol, 2009). Likewise, they also comprise risk factors for age-related neurodegenerative diseases, where they initiate and propagate oxidative damage in the brain (Wadhwa et al., 2018; Kothapalli, 2021; Raj et al., 2021). Interestingly, there was no clear correlation found between increasing age and the concentration of certain heavy metals in the blood of patients with no documented heavy metal exposure. However, bone concentrations of chromium, cobalt, and thallium decreased with increasing age, and there was a close association between cobalt bone concentration and osteopenia, a common disorder in the elderly (Chang et al., 2018). Inversely, the accumulation of heavy metals led to stem cell dysfunctions *via* oxidative damage, eventually affecting the body’ s regenerative ability, the occurrence of age-related diseases, and the premature aging phenotype. This was particularly evident in cadmium-treated pluripotent and adult stem cells (Hussein and Hasan, 2010), as well as in prostate stem progenitor cells (Jiang et al., 2011). Similar pro-oxidant toxicity and dysfunctions were found for mercury-exposed rat neural stem cells (Tamm et al., 2006), and for lead- and arsenic-treated mesenchymal stem cells (Ahmad and Shakoori, 2013).

#### Ultraviolet Radiation

UV radiation can increase oxidative stress by either stimulating the production of ROS or causing immediate DNA damage. This increase in oxidative stress can impair cellular functions, leading to, for example, exogenous skin aging (Rinnerthaler et al., 2015; Naidoo and Birch-Machin, 2017). UV radiation also affects mtDNA. In particular, it causes a deficiency of 4,977 bases in mtDNA, a process that contributes to elevated ROS production in mitochondria and therefore, to the promotion of mitochondrial damage (Berneburg et al., 1997; Rinnerthaler et al., 2015).

Both intrinsic and exogenous aging contribute to skin aging. Exogenous skin aging is caused by chronic overexposure to UV radiation. Accumulated damage caused by UV radiation, especially UVB and UVA, leads to local premature aging of the skin, known as photoaging. Photoaging is characterized by damages in the structure of the epidermis and dermis, hyperpigmentation, sagging, and the formation of wrinkles. The underlying mechanism of photoaging has not been fully understood (Krutmann and Schroeder, 2009; D’Orazio et al., 2013).

In skin cells under the influence of UVB radiation, RNA-dependent protein kinase R (PKR) was found to be phosphorylated by MAPKs kinases. The uncontrolled activity of PKR led to an increase in the expression of pro-inflammatory cytokines, such as MMP-9, collagenase type IV, and cyclooxygenase-2 (COX-2), which together accelerated the inflammatory process and skin aging (Lee et al., 2019).

In another relevant study, it was observed that melanocytes could senescence under UVA and UVB radiation, expressing SASP factors such as p16^INK4a^ and dysfunctional telomeres. These senescent melanocytes impaired keratinocyte proliferation in a ROS-dependent manner leading to skin age-related phenotypes. Both clearance of senescent cells with the drug ABT737 and treatment with the mitochondrial-specific antioxidant MitoQ abrogated this aging effect, further promoting the association between ROS, senescence, and aging in the skin (Victorelli et al., 2019).

#### Air Pollution

Environmental pollution is constantly increasing worldwide and the impact of pollutants on human health is a matter of particular concern. The majority of air pollutants come from anthropogenic sources, such as emissions from motor vehicles, fossil fuel combustion, forest fires, and industrial plants. This variety of sources produces a complex mixture of toxic pollutants, including particulate matter (particle pollution) and gases such as NO_2_ and O_3_ (Krutmann et al., 2014). Particles with a diameter of less than 0.1 μm are emitted from the vehicle exhaust gas. These particles are particularly harmful because of their ability to penetrate tissues more easily and translocate in the mitochondria. Once absorbed, these particles cause oxidative stress and damage to mitochondria. Ozone, an important component of smog, is an extremely active environmental pollutant that can cause oxidative stress, either directly by oxidizing biomolecules to generate ROS or by producing cytotoxic non-radical molecules (Valacchi et al., 2005).

Further to this process, air pollution has also been linked to oxidative stress-induced placental alterations early in the life of embryos, which leads to telomeres shortening (Martens et al., 2017), decrease in mtDNA content (Janssen et al., 2012) and p53- and miRNA-regulated aging phenotype followed by later-life diseases (Zhou et al., 2016; Saenen et al., 2019). Air pollution exposure can provoke nitrosative stress, epigenetic alterations, and increased aging markers. Since small particles can penetrate the placental barrier, oxidative damage can be induced, as shown in the study of Pavel et al. (2011) who showed increased 8-OHdG in the mitochondria of the placenta after air pollution exposure during the first 4 months of pregnancy. Thus, these processes lead to potential implications for the aging phenotype (Martens and Nawrot, 2018).

#### Smoking

Smoking is an environmental factor that puts a lot of pressure on the health system, as it contributes to the development of diseases such as cancer (most likely lung cancer), cardiovascular and respiratory diseases, and premature aging. It is also strongly linked to oxidative stress (Nicita-Mauro et al., 2008a, b; Daskalopoulou et al., 2018; Padmavathi et al., 2018). Indeed, several studies have shown that smoking is associated with a more rapid onset of aging characteristics, such as fragility and mortality risk (Zuo et al., 2014; Liguori et al., 2018). The analysis of DNA methylation was the predominant method for predicting biological age (Bell et al., 2019). Recently, it has been suggested that hematological markers identified in routine testing, such as fasting blood glucose and red cell distribution width, may serve as predictive markers (Mamoshina et al., 2019). Using these markers and predictive aging models, it was observed that smokers had higher aging rates compared to non-smokers, regardless of cholesterol and blood glucose levels. Female smokers were estimated to be twice their biological age compared to their chronological age, whereas male smokers were one and a half times their biological age compared to their actual age compared to non-smokers (Mamoshina et al., 2019).

Furthermore, epidemiological studies have proposed that smoking leads to a fast decline in lung function, suggesting that it increases the age of the lungs. This hypothesis was supported by the analysis of leukocyte telomere length, which showed that smoking was associated with shorter telomeres (Walters et al., 2014). Indeed, another study also associated cellular senescence with lung aging, chronic obstructive pulmonary disease (COPD), and smoke exposure. Patients with COPD had small airway epithelial cells with high telomere-associated DNA damage, similar to aged murine lungs, which was further accelerated by cigarette smoke exposure. Smoking was found to *in vitro* accelerate telomere dysfunction *via* the production of ROS and was also linked to proinflammatory cytokine secretion. Thus, telomere dysfunction could drive lung deterioration found in aging and COPD and was negatively associated with smoke exposure (Birch et al., 2015). In addition, cellular senescence biomarkers, such as p16^Ink4a^, were increased in idiopathic pulmonary fibrosis lung tissue, whereas the removal of senescent cells *via* the co-administration of dasatinib and quercetin helped with the improvement of lung function (Schafer et al., 2017).

Finally, in elderly smokers, serum cotinine levels and the degree of DNA methylation were associated with oxidative stress markers, such as 8-isoprostane (8-iso) and 8-hydroxy-2’-deoxyguanosine (8-oxodG), which were determined in the urine. “Current” smoking, cumulative exposure to smoking (how many packs of cigarettes were used during smoking years), and serum cotinine levels were correlated with urinary 8-iso levels but not with 8-oxodG levels. In addition, 71 cytosine and guanine-rich regions (CpG islands) were found to be associated with smoking and correlated with 8-iso levels. Thus, CpG islands, that are methylation sites, could potentially be an epigenetic marker of the smoking-induced oxidative stress (Gao et al., 2017).

## Factors That Prolong The Onset of The Aging Process

Once the theory that oxidative stress is involved in the aging process has been proposed, it has been concluded that antioxidants could play a protective role in aging. The antioxidant strategy includes reducing the levels of oxidative stress and/or strengthening the antioxidant defense mechanisms. Thus, given the hypothesis that the harmful effects of oxidative stress can be eliminated by antioxidants, many studies have been conducted over the past three decades with the aim to explore the effects of antioxidant strategies on age- and oxidative stress-related diseases. However, no absolute positive correlation has been observed between taking supplements, herbs, and natural products and the deceleration of aging, as previously discussed (Pérez et al., 2009; Ghezzi et al., 2017; Tan et al., 2018). One additional example is the well-known antioxidant vitamin E, for which animal studies have shown that it is a potent antioxidant with good protective activity, but clinical studies have not shown a protective effect in age-related diseases (Devaraj and Jialal, 2005; Banks et al., 2010; Wang et al., 2014; Luo et al., 2020).

At the same time, reducing calorie intake and increasing the activity of SIRT1 could reduce the oxidative load (Grabowska et al., 2017). Moreover, the role of exercise in preventing premature aging has been investigated. Chronic muscular exercise has been found to help older people acquire lower oxidative load, while acute exercise increases ROS production and ultimately causes more damage (Davalli et al., 2016).

### The Effects of Diet on Aging and Antioxidant Compounds With Anti-aging Activity

The effect of diet on oxidative stress has been extensively studied recently, due to the fact that exogenous antioxidants can be absorbed through diet. These compounds can act as a defense mechanism against oxidative stress caused by environmental factors. Mediterranean diet, characterized by a high intake of fruits and vegetables full of antioxidants, has been associated with increased longevity and a reduced risk of age-related diseases (Chrysohoou and Stefanadis, 2013; Vasto et al., 2014; Chatzianagnostou et al., 2015). Studies have proved that adherence to the Mediterranean diet is significantly linked to lower levels of oxidative stress and can prevent cellular aging in ECs (Dai et al., 2008; Marin et al., 2012). In contrast, a diet full of fats has been linked with mitochondrial dysfunction, increased levels of oxidative stress, and accelerated cellular aging (Fujita et al., 2006; de Oliveira et al., 2011; Bonomini et al., 2015). Some of the substances with anti-aging properties contained in the Mediterranean diet are resveratrol, found in grapes and red wine, and quercetin, found in citrus fruits, onions, and red wine (Chatzianagnostou et al., 2015; Maurya et al., 2016).

#### Resveratrol

One of the compounds that activate sirtuins, and in particular the signaling pathway induced by SIRT1, is resveratrol (Howitz et al., 2003). Resveratrol is a polyphenolic molecule found in several natural products, such as red wine, grapes, and peanuts (Wallerath et al., 2002). In recent years, the biological action of resveratrol has been extensively studied. It has been shown to have antioxidant and anti-inflammatory properties, so its use is being investigated in the context of diseases such as type 2 diabetes and cardiovascular diseases (Pearson et al., 2008; Wong et al., 2011; Mattison et al., 2014; Kulkarni and Cantó, 2015).

Activation of the SIRT1 signaling pathway by resveratrol can affect processes such as metabolism, oxidative stress resistance, cell survival, cellular aging, immune function, endothelial cell function, and circadian rhythm (Baur et al., 2006; Murase et al., 2009; Kao et al., 2010; Lin et al., 2014; Ramis et al., 2015). Furthermore, resveratrol was shown to phosphorylate and activate AMPK (5’ adenosine monophosphate-activated protein kinase; Liu et al., 2016). AMPK is an enzyme found in all cells and plays a role in the homeostasis of cellular energy, mainly functioning in order to activate glucose and fatty acid uptake and oxidation when the cellular energy levels are low (Min and Ki, 2005). SIRT1 and AMPK play a similar role, including their ability to respond to oxidative stress, induce mitochondrial biogenesis, regulate glucose homeostasis and control the activity of important transcription factors such as PGC-1a and FOXOs (Fulco and Sartorelli, 2008; Price et al., 2012).

A study in middle-aged and elderly patients found a reduction in ROS in resveratrol-treated peripheral blood mononuclear cells isolated from the patients. However, the following difference was observed between the two age groups: in elderly patients there was a higher production of ROS, and the reduction caused by resveratrol was smaller than in the middle-aged group. In addition, there was increased SOD activity in resveratrol-treated cells in the elderly group. For the middle-aged participants, it was observed that SIRT1 and AMPK participated in antioxidant pathways and that resveratrol acted through SIRT1, whereas in the elderly no similar results were observed (Caldeira et al., 2021).

In another relevant study, researchers investigated the ability of resveratrol to modulate PMRS during aging in 97 healthy humans. It was found that resveratrol upregulated PMRS along with ascorbate free radical reductase, protected against lipid peroxidation and protein carbonylation, and restored the levels of GSH and -SH groups during an oxidative stress-induced injury in erythrocytes of all age groups (young, middle-aged, old). Thus, this study underlined the importance of ascorbic acid regeneration and its role as a primary plasma antioxidant that is upregulated by resveratrol (Pandey and Rizvi, 2013).

#### Quercetin

Quercetin is one of the most common flavonoids in our diet, as it is included in a wide range of foods, such as grape peel, red onion, green tea, or tomatoes (Li et al., 2016). Quercetin is of great interest to scientists because of its unique anti-aging properties (Chondrogianni et al., 2010). Quercetin belongs to the senolytics, a group of drugs which selectively kill senescent cells.

Recently, the mechanism by which quercetin reduces age-related neurological disorders has been described. Quercetin was administered orally to 7-month-old mice in two doses, 35 and 70 mg/kg for 4 weeks. Behavioral experiments showed that the mice, at the end of the quercetin intervention, showed improved spatial learning and memory. In order to investigate the molecular mechanism of this effect, mice hippocampus was isolated and the expression of SIRT1, inflammasome proteins such as NLRP3 (NLR family pyrin domain containing 3) and ASC (apoptosis-associated speck-like protein), the synaptic marker PSD95 (postsynaptic density protein 95), and the neutrophic factors BDNF (brain-derived neurotrophic factor) and NGF (nerve growth factor) was measured. The results showed that quercetin intervention increased the expression of SIRT1 and prevented neuroinflammation, a finding based on the decreased protein expression of the astrocyte marker GFAP (glial fibrillary acidic protein, neurological damage marker), and proinflammatory factors such as IL-1β, caspase 1, and IL-18. Moreover, quercetin reduced the levels of malondialdehyde and ROS in the hippocampus of the elderly mice (Li et al., 2021).

In a previously described study, idiopathic pulmonary fibrosis lung tissue expressed several cellular senescence biomarkers, but the treatment with dasatinib and quercetin eliminated senescent cells and ameliorated lung function (Schafer et al., 2017). Similarly, dasatinib and quercetin were used in another study where they were found to successfully eliminate senescent cells *in vitro*, but most importantly they also led to a marked decrease in senescent cells in aged, radiation-exposed, and progeroid mice *in vivo*. Several functions of the aged mice, such as cardiac function, were improved even after a single dose, whereas treated mice with one irradiated limb were more capable of exercising. Finally, progeroid mice benefited from an extended healthspan and delayed age-related phenotype and relevant pathologies (Zhu et al., 2015).

Moreover, another *in vivo* study of the combination of dasatinib with quercetin showed reduced intervertebral disc degeneration, which is associated with chronic back pain and disability frequently observed in the elderly. These senolytic drugs decreased senescence marker expression and preserved cell viability, phenotype, and matrix content, resulting in alleviated age-related disc degeneration (Novais et al., 2021). Lastly, a Phase I pilot study was designed for the oral co-treatment of dasatinib (100 mg) and quercetin (1,000 mg) for 3 days in elderly patients with diabetic kidney disease. The treatment reduced adipose tissue senescent cells and the chemotaxis of adipose tissue macrophages within 11 days. Several senescence and SASP factors were found downregulated, including IL-1α and IL-6 among others. Therefore, the short-term treatment with senolytic drugs reduced senescent cell burden in humans, suggesting the anti-aging activity of these drugs (Hickson et al., 2019).

#### Vitamin D

Vitamin D can enhance cognitive functions, slow down aging and protect humans from age-related diseases (Bartali et al., 2014; Yang et al., 2020). Vitamin D can successfully affect the mechanisms that regulate aging in humans, a fact that has redirected the interest of the scientific community on its potential anti-aging properties (Chan and Woo, 2011; Meehan and Penckofer, 2014; Bocheva et al., 2021).

Steadily increasing findings show that vitamin D has antioxidant properties (Sepidarkish et al., 2019). In a study of 302 participants aged 62–92 years old, serum 25-hydroxyvitamin D levels were quantified and correlated with the overall redox status and antioxidant enzymes such as CAT, GPx, and SOD. The results of the study showed that 25-hydroxyvitamin D was positively correlated with the expression of antioxidant enzymes, indicating that adequate levels of vitamin D strengthen the antioxidant defense (Jungert and Neuhäuser-Berthold, 2018).

### The Role of Exercise and the Reduction of Calorie Intake as Anti-aging Strategies

Physical activity significantly contributes to having a healthy, pleasant, and long life. Numerous studies have shown that there is an inextricable link and interaction between exercise, fitness, and human health (Farris and Abrantes, 2020; Raza et al., 2020; Maynou et al., 2021).

A recent study aimed to compare two different types of exercise not only in physical condition but also in the levels of oxidative and inflammatory factors. Specifically, 36 middle-aged female rats (18 months old) underwent either 8 weeks of moderate intensity of continuous exercise or high intensity of intermittent exercise (45 min, 5 times a week). The results were compared with control rats of the same age that did not follow an exercise program. Subsequently, the effects of these exercise programs on physical condition, levels of inflammatory factors, adipocytokines (leptin, adiponectin) in adipose tissue and blood serum, as well as oxidative stress markers, IGF-1, blood glucose, and the rats’ lipidemic profiles were investigated. Animals’ weight and the fat percentage increased from 18 to 26 months as a result of aging, while only intermittent exercise effectively reduced animals’ weight. In addition, intermittent exercise improved animals’ strength and endurance. Rats following the intermittent exercise program expressed reduced levels of C-reactive protein (CRP) and higher serum IL-10 levels, compared with control rats and those who exercised continuously. Both types of exercise increased IGF-1 in skeletal muscles and decreased its serum levels, while also reducing adiponectin levels. Rats at 26 months of age had elevated levels of free fatty acids, but intermittent exercise decreased these levels and increased leptin in the serum while decreasing it in the adipose tissue. Intermittent exercise also reduced malondialdehyde levels in the serum and skeletal muscles compared with continuous exercise. Both types of exercise resulted in a similar decrease in serum and skeletal muscle levels of 4-hydroxynonenal and 8-oxodG in the serum, while they also increased the activity of the antioxidant enzyme SOD-2. In conclusion, intermittent exercise, even when adopted in middle age, has beneficial effects on oxidative and inflammatory factors that increase with age and contribute to aging (Li et al., 2018).

Reducing calorie intake is an invasive strategy that has been shown to have beneficial effects on the lifespan of several animals (Miller et al., 2005; Sun et al., 2009; Colman et al., 2014), although similar results were reported for dietary restriction without caloric restriction (CR; Lee and Longo, 2016). The mechanism underlying this beneficial action remains under investigation, but it seems that the positive effects are associated, among others, with the cellular redox state.

Indeed, CR was shown to protect the brain against aging and related diseases by increasing the activity of multiple PMRS enzymes, such as NADH-ascorbate free radical reductase, and the levels of antioxidants, such as α-tocopherol and coenzyme Q10 in neuronal plasma membranes during aging. Moreover, a decrease in age-driven plasma membrane lipid peroxidation, protein carbonylation, and nitrotyrosine were observed after CR, hence downregulating the biomarkers of oxidative stress (Hyun B.-H. et al., 2006).

Moreover, moderate CR prevented the age-induced increase of oxidative stress in cerebromicrovascular ECs (CMVECs), while this activity was linked to the restoration of Nrf-2 action and miR-144 expression. Although miR-144 was found upregulated in aged CMVECs and Nrf-2 was downregulated, CR was able to revert these changes, also leading to anti-inflammatory, antioxidant and pro-angiogenic effects, as well as to the evasion of the age-related increase of NF-κB and pro-inflammatory shift in the secretome of ECs, which are critical for EC homeostasis. These results were also consistent with the *in vivo* findings, further contributing to the miRNA-dependent CR effects against aging (Csiszar et al., 2014).

However, in an experimental study using transgenic mice models lacking the *sirtuin 3 (SIRT3)* gene, it was observed that these mice had increased oxidative stress and therefore increased oxidative damage, despite CR. This finding highlighted the role of SIRT3 along with CR, which have been shown to reduce cellular ROS levels. SIRT3 deacetylates two important lysine residues in SOD2, promoting its antioxidant activity (Qiu et al., 2010).

## Discussion and Future Prospect

The discovery that ROS is responsible for cellular oxidative damage and is associated with age-related degenerative diseases has been a key contributor to making the oxidative stress theory one of the leading theories in the pathophysiology of aging, even with exceptions (Kirkwood, 2005b). The molecular mechanisms underlying oxidative stress signaling and its impact on age-related pathologies such as cardiovascular diseases, diabetes and obesity have been extensively reviewed over the years (Matsuda and Shimomura, 2013; Nijhawan et al., 2019; Tisi et al., 2021; Alvarado et al., 2022; Hajam et al., 2022). However, the implication of oxidative stress in aging and SASP factors has been better elucidated in this review, while particular mention was made to exogenous factors that can affect aging through the production of ROS, further highlighting the importance of this mechanism towards the complete explanation of the aging phenomenon.

So, the question arises; How can we delay the onset and progression of aging? Despite the comprehensive studies that largely deciphered the oxidative stress-related molecular mechanisms involved in aging, there is an ongoing controversy over antioxidant supplementation for the prevention or even therapy of aging and age-related diseases. It seems that several antioxidant compounds have conferred many desired anti-aging effects, although not reaching a favorable conclusive endpoint in clinical trials, for example, vitamin E or resveratrol. Additionally, even though in studies with model organisms the maximum lifespan could be increased, the maximum lifespan of humans has not really changed since the 1990s. This may propose that the maximum lifespan of humans has already been reached, is fixed, and subject to natural constraints. Of note, multifactor nutritional treatments may be the answer to this question, i.e., combining an antioxidant compound with agents that directly affect mediators of aging, such as SIRT1 activators or NAD^+^supplementation or silencing of miRNAs related to this process, which remain to be explored. Other therapeutic strategies to curb the effects of oxidative stress include caloric restriction and intermittent exercise, which have been shown to ameliorate senescence biomarkers, such as SA-β-GAL, p16 (p16^Ink4a^), p21, p53, and inflammatory cytokines (IL-6, IL-8), and even the extent the lifespan of animal models. As for caloric restriction, it remains to be explored if just dietary restrictions would have the same beneficial effects, by determining which key factors contribute to the anti-aging results.

However, there could be no effective anti-aging strategy without first accrediting the environmental stressors that further add to the oxidative load and contribute to faster aging. Continuous exposure to heavy metals, air pollution, or UV radiation has a significant impact on the molecular mechanisms involved, hence a successful anti-aging therapy would have to protect the organism from such detrimental impacts. Smoking has been conclusively linked to faster organ aging and higher aging rates, provoking several oxidative genetic and epigenetic changes, so again eliminating this factor can contribute to healthier aging. Therefore, it seems that reducing the oxidative load with combination dietary therapies alone is not enough, and healthy or at least delayed aging should also be accompanied by radical changes in lifestyle and daily habits.

## Conclusion

In conclusion, this literature review presented extended scientific data on the biochemical and molecular mechanisms of aging and deciphered the involvement of oxidative stress in this process. The combination of experimental, epidemiological, and clinical studies presented here has expanded our knowledge of this field and proved that promising therapies against age-related diseases involve various antioxidant agents. Indeed, by attenuating ROS-activated signaling pathways we could delay the expression of SASP factors and the accumulation of senescent cells, key features of aging tissues. Moreover, through this review, specific environmental and lifestyle-related factors that enhance oxidative stress and become leading causes of aging were highlighted. Thus, a better understanding and detailed assessment of the environmental factors that lead to premature aging, as well as their possible interaction with genetic elements, could contribute to the development of more effective and targeted strategies in order to significantly slow down the onset and progression of the aging process.

## Author Contributions

All authors contributed to the article and approved the submitted version.

## Conflict of Interest

The authors declare that the research was conducted in the absence of any commercial or financial relationships that could be construed as a potential conflict of interest.

## Publisher’s Note

All claims expressed in this article are solely those of the authors and do not necessarily represent those of their affiliated organizations, or those of the publisher, the editors and the reviewers. Any product that may be evaluated in this article, or claim that may be made by its manufacturer, is not guaranteed or endorsed by the publisher.

## Abbreviations

AKT: Protein kinase B
AMPK: 5’ adenosine monophosphate-activated protein kinase
ASC: Apoptosis-associated speck-like protein
ATP: Adenosine triphosphate
BDNF: Brain-derived neurotrophic factor
BMAL1: Brain and muscle aryl hydrocarbon receptor nuclear translocator (ARNT)-like 1
CAT: Catalase
Ccna2: Cyclin-A2
Cdc6: Cell division cycle 6
CMVECs: Cerebromicrovascular endothelial cells
COPD: Chronic obstructive pulmonary disease
COX-2: Cyclooxygenase-2
CR: Caloric restriction
CRP: C-reactive protein
DDR: DNA damage response
DNA: Deoxyribonucleic acid
EC: Endothelial cell
ECM: Extracellular matrix
EMT: Epithelial-mesenchymal transition
ER: Endoplasmic reticulum
FADH_2_: Flavin adenine dinucleotide
FOXO: Forkhead box
GFAP: Glial fibrillary acidic protein
GPx: Glutathione peroxidase
GSH: Glutathione
IFN-γ: Interferon γ
IGF-1: Insulin-like growth factor 1
IL: Interleukin
INPP4A: Inositol polyphosphate-4-phosphatase type I A
ISS: Insulin/IGF-1 signaling pathway
miRNA: MicroRNA
MMPs: Matrix metalloproteinases
mtDNA: Mitochondrial DNA
mTOR: Mechanistic/Mammalian target of rapamycin
mTORC1/mTORC2: Mammalian target of rapamycin complex 1/2
NADH: Nicotinamide adenine dinucleotide
NADPH: Nicotinamide adenine dinucleotide phosphate
NF-κB: Nuclear factor kappa-light-chain-enhancer of activated B cells
NF1: Neurofibromin 1
NGF: Nerve growth factor
NLRP3: NOD-like receptor family pyrin domain containing 3
NOX: NADPH oxidase
Nrf-2: Nuclear factor erythroid 2–related factor 2
p38MAPK: p38 mitogen-activated protein kinase
PD-1: Programmed cell death-1
PGC-1a: Peroxisome proliferator-activated receptor gamma coactivator 1-alpha
PKR: Protein kinase RNA-activated
PMRS: Plasma membrane redox system
PSD95: Postsynaptic density protein 95
PTEN: Phosphatase and tensin homolog
RB1: Retinoblastomaprotein1
RCS: Reactive chlorine species
RNS: Reactive nitrogen species
ROS: Reactive oxygen species
SA-β-GAL: Senescence-associated beta-galactosidase
SASP: Senescence-associated secretory phenotype
SERCA: Sarco/endoplasmic reticulum Ca^2+^-ATPase
SGK: Serine/threonine-protein kinases
SIRT1–7: Sirtuin 1–7
SOD: Superoxide dismutase
SR: Sarcoplasmic reticulum
Tfam: Mitochondrial transcription factor A
TFEB: Transcription factor EB
TIMPs: Tissue inhibitors of metalloproteinases
TNF-α: Tumor necrosis factor α
UV: Ultraviolet radiation.

## References

[B1] Aboelella N. S. Brandle C. Kim T. Ding Z.-C. Zhou G. (2021). Oxidative stress in the tumor microenvironment and its relevance to cancer immunotherapy. Cancers (Basel) 13:986. 10.3390/cancers13050986 33673398 PMC7956301

[B2] Acosta J. C. Banito A. Wuestefeld T. Georgilis A. Janich P. Morton J. P.. (2013). A complex secretory program orchestrated by the inflammasome controls paracrine senescence. Nat. Cell Biol. 15, 978–990. 10.1038/ncb2784 23770676 PMC3732483

[B3] Acosta J. C. O’Loghlen A. Banito A. Guijarro M. V. Augert A. Raguz S.. (2008). Chemokine signaling via the CXCR2 receptor reinforces senescence. Cell 133, 1006–1018. 10.1016/j.cell.2008.03.038 18555777

[B4] Ahmad A. Shakoori A. R. (2013). Cytotoxic and genotoxic effects of arsenic and lead on human adipose derived mesenchymal stem cells (AMSCs). J. Stem Cells Regen. Med. 9, 29–36. 10.46582/jsrm.0902007 24693207 PMC3908312

[B5] Aird K. M. Zhang G. Li H. Tu Z. Bitler B. G. Garipov A.. (2013). Suppression of nucleotide metabolism underlies the establishment and maintenance of oncogene-induced senescence. Cell Rep. 3, 1252–1265. 10.1016/j.celrep.2013.03.004 23562156 PMC3840499

[B7] Alvarado J. C. Fuentes-Santamaría V. Juiz J. M. (2022). Frailty syndrome and oxidative stress as possible links between age-related hearing loss and Alzheimer’s disease. Front. Neurosci. 15:816300. 10.3389/fnins.2021.816300 35115905 PMC8804094

[B8] Amaro-Ortiz A. Yan B. D’Orazio J. A. (2014). Ultraviolet radiation, aging and the skin: prevention of damage by topical cAMP manipulation. Molecules 19, 6202–6219. 10.3390/molecules19056202 24838074 PMC4344124

[B9] Anisimov V. N. Zabezhinski M. A. Popovich I. G. Piskunova T. S. Semenchenko A. V. Tyndyk M. L.. (2011). Rapamycin increases lifespan and inhibits spontaneous tumorigenesis in inbred female mice. Cell Cycle 10, 4230–4236. 10.4161/cc.10.24.18486 22107964

[B10] Baar M. P. Brandt R. M. C. Putavet D. A. Klein J. D. D. Derks K. W. J. Bourgeois B. R. M.. (2017). Targeted apoptosis of senescent cells restores tissue homeostasis in response to chemotoxicity and aging. Cell 169, 132–147.e16. 10.1016/j.cell.2017.02.031 28340339 PMC5556182

[B11] Babušíková E. Lehotský J. Dobrota D. Račay P. Kaplán P. (2012). Age-associated changes in Ca^2+^-atpase and oxidative damage in sarcoplasmic reticulum of rat heart. Physiol. Res. 61, 453–460. 10.33549/physiolres.932320 22881224

[B12] Baker D. J. Jeganathan K. B. Cameron J. D. Thompson M. Juneja S. Kopecka A.. (2004). BubR1 insufficiency causes early onset of aging-associated phenotypes and infertility in mice. Nat. Genet. 36, 744–749. 10.1038/ng1382 15208629

[B13] Baker D. J. Wijshake T. Tchkonia T. Lebrasseur N. K. Childs B. G. van de Sluis B.. (2011). Clearance of p16 Ink4a-positive senescent cells delays ageing-associated disorders. Nature 479, 232–236. 10.1038/nature10600 22048312 PMC3468323

[B14] Banan A. Fields J. Z. Zhang Y. Keshavarzian A. (2001). Phospholipase C-γ inhibition prevents EGF protection of intestinal cytoskeleton and barrier against oxidants. Am. J. Physiol. Gastrointest. Liver Physiol. 281, G412–G423. 10.1152/ajpgi.2001.281.2.G412 11447022

[B15] Bandy B. Davison A. J. (1990). Mitochondrial mutations may increase oxidative stress: implications for carcinogenesis and aging? Free Radic. Biol. Med. 8, 523–539. 10.1016/0891-5849(90)90152-9 2193852

[B16] Banks R. Speakman J. R. Selman C. (2010). Vitamin E supplementation and mammalian lifespan. Mol. Nutr. Food Res. 54, 719–725. 10.1002/mnfr.200900382 20205192

[B17] Barascu A. le Chalony C. Pennarun G. Genet D. Imam N. Lopez B.. (2012). Oxidative stress induces an ATM-independent senescence pathway through p38 MAPK-mediated lamin B1 accumulation. EMBO J. 31, 1080–1094. 10.1038/emboj.2011.492 22246186 PMC3297999

[B18] Bartali B. Devore E. Grodstein F. Kang J. H. (2014). Plasma vitamin D levels and cognitive function in aging women: the nurses’ health study. J. Nutr. Health Aging 18, 400–406. 10.1007/s12603-013-0409-9 24676321 PMC4198067

[B19] Bates D. J. Li N. Liang R. Sarojini H. An J. Masternak M. M.. (2010). MicroRNA regulation in Ames dwarf mouse liver may contribute to delayed aging. Aging Cell 9, 1–18. 10.1111/j.1474-9726.2009.00529.x 19878148 PMC2844644

[B20] Baur J. A. Pearson K. J. Price N. L. Jamieson H. A. Lerin C. Kalra A.. (2006). Resveratrol improves health and survival of mice on a high-calorie diet. Nature 444, 337–342. 10.1038/nature05354 17086191 PMC4990206

[B21] Bautista-Niño P. K. Portilla-Fernandez E. Vaughan D. E. Danser A. H. J. Roks A. J. M. (2016). DNA damage: a main determinant of vascular aging. Int. J. Mol. Sci. 17:748. 10.3390/ijms17050748 27213333 PMC4881569

[B22] Bell C. G. Lowe R. Adams P. D. Baccarelli A. A. Beck S. Bell J. T.. (2019). DNA methylation aging clocks: challenges and recommendations. Genome Biol. 20:249. 10.1186/s13059-019-1824-y 31767039 PMC6876109

[B23] Benkafadar N. François F. Affortit C. Casas F. Ceccato J.-C. Menardo J.. (2019). ROS-induced activation of DNA damage responses drives senescence-like state in postmitotic cochlear cells: implication for hearing preservation. Mol. Neurobiol. 56, 5950–5969. 10.1007/s12035-019-1493-6 30693443 PMC6614136

[B24] Berneburg M. Gattermann N. Stege H. Grewe M. Vogelsang K. Ruzicka T.. (1997). Chronically ultraviolet-exposed human skin shows a higher mutation frequency of mitochondrial DNA as compared to unexposed skin and the hematopoietic system. Photochem. Photobiol. 66, 271–275. 10.1111/j.1751-1097.1997.tb08654.x 9277148

[B25] Bhaskar P. T. Hay N. (2007). The two TORCs and Akt. Dev. Cell 12, 487–502. 10.1016/j.devcel.2007.03.020 17419990

[B26] Bhaumik D. Scott G. K. Schokrpur S. Patil C. K. Orjalo A. V. Rodier F.. (2009). MicroRNAs miR-146a/b negatively modulate the senescence-associated inflammatory mediators IL-6 and IL-8. Aging (Albany NY) 1, 402–411. 10.18632/aging.100042 20148189 PMC2818025

[B27] Birch J. Anderson R. K. Correia-Melo C. Jurk D. Hewitt G. Madeira Marques F.. (2015). DNA damage response at telomeres contributes to lung aging and chronic obstructive pulmonary disease. Am. J. Physiol. Lung Cell. Mol. Physiol. 309, L1124–L1137. 10.1152/ajplung.00293.2015 26386121 PMC4652155

[B28] Birch J. Passos J. F. (2017). Targeting the SASP to combat ageing: mitochondria as possible intracellular allies? Bioessays 39:1600235. 10.1002/bies.201600235 28217839

[B29] Birch-Machin M. A. Swalwell H. (2010). How mitochondria record the effects of UV exposure and oxidative stress using human skin as a model tissue. Mutagenesis 25, 101–107. 10.1093/mutage/gep061 19955330

[B30] Blackburn E. H. Epel E. S. Lin J. (2015). Human telomere biology: a contributory and interactive factor in aging, disease risks and protection. Science 350, 1193–1198. 10.1126/science.aab3389 26785477

[B31] Bleier L. Wittig I. Heide H. Steger M. Brandt U. Dröse S. (2015). Generator-specific targets of mitochondrial reactive oxygen species. Free Radic. Biol. Med. 78, 1–10. 10.1016/j.freeradbiomed.2014.10.511 25451644

[B32] Bocheva G. Slominski R. M. Slominski A. T. (2021). The impact of vitamin D on skin aging. Int. J. Mol. Sci. 22:9097. 10.3390/ijms22169097 34445803 PMC8396468

[B33] Bodnar A. G. Ouellette M. Frolkis M. Holt S. E. Chiu C.-P. Morin G. B.. (1998). Extension of life-span by introduction of telomerase into normal human cells. Science 279, 349–352. 10.1126/science.279.5349.349 9454332

[B34] Boehm M. Slack F. (2005). A developmental timing microRNA and its target regulate life span in *C. elegans*. Science 310, 1954–1957. 10.1126/science.1115596 16373574

[B35] Bohr V. A. (2002). Repair of oxidative DNA damage in nuclear and mitochondrial DNA and some changes with aging in mammalian cells. Free Radic. Biol. Med. 32, 804–812. 10.1016/s0891-5849(02)00787-6 11978482

[B36] Bonomini F. Rodella L. F. Rezzani R. (2015). Metabolic syndrome, aging and involvement of oxidative stress. Aging Dis. 6, 109–120. 10.14336/AD.2014.0305 25821639 PMC4365955

[B37] Brainina K. Stozhko N. Vidrevich M. (2019). Antioxidants: terminology, methods and future considerations. Antioxidants (Basel) 8:297. 10.3390/antiox8080297 31404992 PMC6720181

[B38] Brown G. C. (2015). Living too long: the current focus of medical research on increasing the quantity, rather than the quality, of life is damaging our health and harming the economy. EMBO Rep. 16, 137–141. 10.15252/embr.201439518 25525070 PMC4328740

[B39] Budanov A. V. (2014). “The role of tumor suppressor p53 in the antioxidant defense and metabolism,” in Mutant p53 and MDM2 in Cancer. Subcellular Biochemistry, Vol. 85., eds Deb S. Deb S. (Dordrecht: Springer), 337–358. 10.1007/978-94-017-9211-0_18 PMC4206257 25201203

[B40] Bu H. Baraldo G. Lepperdinger G. Jansen-Dürr P. (2016). mir-24 activity propagates stress-induced senescence by down regulating DNA topoisomerase 1. Exp. Gerontol. 75, 48–52. 10.1016/j.exger.2015.12.012 26748253

[B41] Bunz F. Dutriaux A. Lengauer C. Waldman T. Zhou S. Brown J. P.. (1998). Requirement for p53 and p21 to sustain G2 arrest after DNA damage. Science 282, 1497–1501. 10.1126/science.282.5393.1497 9822382

[B42] Cabral-Pacheco G. A. Garza-Veloz I. Rosa C. C. Ramirez-Acuña J. M. Perez-Romero B. A. Guerrero-Rodriguez J. F.. (2020). The roles of matrix metalloproteinases and their inhibitors in human diseases. Int. J. Mol. Sci. 21:9739. 10.3390/ijms21249739 33419373 PMC7767220

[B43] Caldeira C. A. Santos M. A. Araújo G. R. Lara R. C. Franco F. N. Chaves M. M. (2021). Resveratrol: change of SIRT 1 and AMPK signaling pattern during the aging process. Exp. Gerontol. 146:111226. 10.1016/j.exger.2021.111226 33444643

[B44] Carocho M. Ferreira I. C. F. R. Morales P. Sokovi M. (2018). Antioxidants and prooxidants: effects on health and aging. Oxid. Med. Cell. Longev. 2018:1472708. 10.1155/2018/1472708 29861825 PMC5971338

[B45] Cattan V. Mercier N. Gardner J. P. Regnault V. Labat C. Mäki-Jouppila J.. (2008). Chronic oxidative stress induces a tissue-specific reduction in telomere length in CAST/Ei mice. Free Radic. Biol. Med. 44, 1592–1598. 10.1016/j.freeradbiomed.2008.01.007 18249196

[B47] Cencioni C. Spallotta F. Martelli F. Valente S. Mai A. Zeiher A. M.. (2013). Oxidative stress and epigenetic regulation in ageing and age-related diseases. Int. J. Mol. Sci. 14, 17643–17663. 10.3390/ijms140917643 23989608 PMC3794746

[B48] Chance B. Sies H. Boveris A. (1979). Hydroperoxide metabolism in mammalian organs. Physiol. Rev. 59, 527–605. 10.1152/physrev.1979.59.3.527 37532

[B49] Chandel N. S. Trzyna W. C. McClintock D. S. Schumacker P. T. (2000). Role of oxidants in NF-κB activation and TNF-α gene transcription induced by hypoxia and endotoxin. J. Immunol. 165, 1013–1021. 10.4049/jimmunol.165.2.1013 10878378

[B50] Chang L. Shen S. Zhang Z. Song X. Jiang Q. (2018). Study on the relationship between age and the concentrations of heavy metal elements in human bone. Ann. Transl. Med. 6:320. 10.21037/atm.2018.08.09 30363972 PMC6186975

[B51] Chan R. Woo J. (2011). The value of vitamin D supplementation in older people. Nutr. Ther. Metabol. 29, 8–21.

[B52] Chapman J. Fielder E. Passos J. F. (2019). Mitochondrial dysfunction and cell senescence: deciphering a complex relationship. FEBS Lett. 593, 1566–1579. 10.1002/1873-3468.13498 31211858

[B53] Chapman J. Ng Y. S. Nicholls T. J. (2020). The maintenance of mitochondrial DNA integrity and dynamics by mitochondrial membranes. Life (Basel) 10:164. 10.3390/life10090164 32858900 PMC7555930

[B54] Chatzianagnostou K. del Turco S. Pingitore A. Sabatino L. Vassalle C. (2015). The Mediterranean lifestyle as a non-pharmacological and natural antioxidant for healthy aging. Antioxidants (Basel) 4, 719–736. 10.3390/antiox4040719 26783955 PMC4712942

[B55] Chelikani P. Fita I. Loewen P. C. (2004). Diversity of structures and properties among catalases. Cell. Mol. Life Sci. 61, 192–208. 10.1007/s00018-003-3206-5 14745498 PMC11138816

[B57] Chen J. Huang X. Halicka D. Brodsky S. Avram A. Eskander J.. (2006). Contribution of p16INK4a and p21CIP1 pathways to induction of premature senescence of human endothelial cells: permissive role of p53. Am. J. Physiol. Heart Circ. Physiol. 290, H1575–H1586. 10.1152/ajpheart.00364.2005 16243918

[B59] Chen X. Liang H. van Remmen H. Vijg J. Richardson A. (2004). Catalase transgenic mice: characterization and sensitivity to oxidative stress. Arch. Biochem. Biophys. 422, 197–210. 10.1016/j.abb.2003.12.023 14759608

[B58] Chen Q. M. Prowse K. R. Tu V. C. Purdom S. Linskens M. H. K. (2001). Uncoupling the senescent phenotype from telomere shortening in hydrogen peroxide-treated fibroblasts. Exp. Cell Res. 265, 294–303. 10.1006/excr.2001.5182 11302695

[B56] Chen H.-Z. Wan Y.-Z. Liu D.-P. (2013). Cross-talk between SIRT1 and p66Shc in vascular diseases. Trends Cardiovasc. Med. 23, 237–241. 10.1016/j.tcm.2013.01.001 23499302

[B60] Chinnery P. F. Hudson G. (2013). Mitochondrial genetics. Br. Med. Bull. 106, 135–159. 10.1093/bmb/ldt017 23704099 PMC3675899

[B61] Chondrogianni N. Kapeta S. Chinou I. Vassilatou K. Papassideri I. Gonos E. S. (2010). Anti-ageing and rejuvenating effects of quercetin. Exp. Gerontol. 45, 763–771. 10.1016/j.exger.2010.07.001 20619334

[B62] Chouchani E. T. Pell V. R. Gaude E. Aksentijević D. Sundier S. Y. Robb E. L.. (2014). Ischaemic accumulation of succinate controls reperfusion injury through mitochondrial ROS. Nature 515, 431–435. 10.1038/nature13909 25383517 PMC4255242

[B63] Chrysohoou C. Stefanadis C. (2013). Longevity and diet. Myth or pragmatism? Maturitas 76, 303–307. 10.1016/j.maturitas.2013.09.014 24210636

[B64] Clegg A. Young J. Iliffe S. Rikkert M. O. Rockwood K. (2013). Frailty in elderly people. Lancet 381, P 752–762. 10.1016/S0140-6736(12)62167-9 23395245 PMC4098658

[B65] Colman R. J. Beasley T. M. Kemnitz J. W. Johnson S. C. Weindruch R. Anderson R. M. (2014). Caloric restriction reduces age-related and all-cause mortality in rhesus monkeys. Nat. Commun. 5:3557. 10.1038/ncomms4557 24691430 PMC3988801

[B66] Coppé J.-P. Patil C. K. Rodier F. Krtolica A. Beauséjour C. M. Parrinello S.. (2010). A human-like senescence-associated secretory phenotype is conserved in mouse cells dependent on physiological oxygen. PLoS One 5:e9188. 10.1371/journal.pone.0009188 20169192 PMC2820538

[B67] Coppé J. P. Patil C. K. Rodier F. Sun Y. Muñoz D. P. Goldstein J.. (2008). Senescence-associated secretory phenotypes reveal cell-nonautonomous functions of oncogenic RAS and the p53 tumor suppressor. PLoS Biol. 6, 2853–2868. 10.1371/journal.pbio.0060301 19053174 PMC2592359

[B69] Correia-Melo C. Birch J. Fielder E. Rahmatika D. Taylor J. Chapman J.. (2019). Rapamycin improves healthspan but not inflammaging in *nfκ*b1^−/−^ mice. Aging Cell 18:e12882. 10.1111/acel.12882 30468013 PMC6351839

[B70] Correia-Melo C. Marques F. D. M. Anderson R. Hewitt G. Hewitt R. Cole J.. (2016). Mitochondria are required for pro-ageing features of the senescent phenotype. EMBO J. 35, 724–742. 10.15252/embj.201592862 26848154 PMC4818766

[B71] Cosín-Tomás M. Álvarez-López M. J. Companys-Alemany J. Kaliman P. González-Castillo C. Ortuño-Sahagún D.. (2018). Temporal integrative analysis of mRNA and microRNAs expression profiles and epigenetic alterations in female SAMP8, a model of age-related cognitive decline. Front. Genet. 9:596. 10.3389/fgene.2018.00596 30619445 PMC6297390

[B72] Csiszar A. Gautam T. Sosnowska D. Tarantini S. Banki E. Tucsek Z.. (2014). Caloric restriction confers persistent anti-oxidative, pro-angiogenic and anti-inflammatory effects and promotes anti-aging miRNA expression profile in cerebromicrovascular endothelial cells of aged rats. Am. J. Physiol. Heart Circ. Physiol. 307, H292–H306. 10.1152/ajpheart.00307.2014 24906921 PMC4121647

[B73] Czerska M. Mikołajewska K. Zieliński M. Gromadzińska J. Wąsowicz W. (2015). Today’s oxidative stress markers. Med. Pr. 66, 393–405. 10.13075/mp.5893.00137 26325052

[B75] Dai J. Chen H. Chai Y. (2019). Advanced glycation end products (AGEs) induce apoptosis of fibroblasts by activation of NLRP3 inflammasome via reactive oxygen species (ROS) signaling pathway. Med. Sci. Monit. 25, 7499–7508. 10.12659/MSM.915806 31587010 PMC6792499

[B74] Dai D.-F. Chen T. Johnson S. C. Szeto H. Rabinovitch P. S. (2012). Cardiac aging: from molecular mechanisms to significance in human health and disease. Antioxid. Redox Signal. 16, 1492–1536. 10.1089/ars.2011.4179 22229339 PMC3329953

[B76] Dai J. Jones D. P. Goldberg J. Ziegler T. R. Bostick R. M. Wilson P. W.. (2008). Association between adherence to the Mediterranean diet and oxidative stress. Am. J. Clin. Nutr. 88, 1364–1370. 10.3945/ajcn.2008.26528 18996873 PMC3076211

[B77] Dasgupta J. Kar S. Liu R. Joseph J. Kalyanaraman B. Remington S. J.. (2010). Reactive oxygen species control senescence-associated matrix metalloproteinase-1 through c-Jun-N-terminal kinase. J. Cell. Physiol. 225, 52–62. 10.1002/jcp.22193 20648623 PMC2913426

[B78] Daskalopoulou C. Stubbs B. Kralj C. Koukounari A. Prince M. Prina A. M. (2018). Associations of smoking and alcohol consumption with healthy ageing: a systematic review and meta-analysis of longitudinal studies. BMJ Open 8:e019540. 10.1136/bmjopen-2017-019540 29666127 PMC5905752

[B79] Das K. C. Muniyappa H. (2013). Age-dependent mitochondrial energy dynamics in the mice heart: role of superoxide dismutase-2. Exp. Gerontol. 48, 947–959. 10.1016/j.exger.2013.06.002 23806974 PMC4045457

[B80] Davaapil H. Brockes J. P. Yun M. H. (2017). Conserved and novel functions of programmed cellular senescence during vertebrate development. Development 144, 106–114. 10.1242/dev.138222 27888193 PMC5278627

[B81] Davalli P. Mitic T. Caporali A. Lauriola A. D’Arca D. (2016). ROS, cell senescence and novel molecular mechanisms in aging and age-related diseases. Oxid. Med. Cell. Longev. 2016:3565127. 10.1155/2016/3565127 27247702 PMC4877482

[B82] Davalos A. R. Coppe J.-P. Campisi J. Desprez P.-Y. (2010). Senescent cells as a source of inflammatory factors for tumor progression. Cancer Metastasis Rev. 29, 273–283. 10.1007/s10555-010-9220-9 20390322 PMC2865636

[B6] de Almeida A. J. P. O. de Almeida Rezende M. S. Dantas S. H. de Lima Silva S. de Oliveira J. C. P. L. de Lourdes Assunção Araújo De Azevedo F.. (2020). Unveiling the role of inflammation and oxidative stress on age-related cardiovascular diseases. Oxid. Med. Cell. Longev. 2020:1954398. 10.1155/2020/1954398 32454933 PMC7232723

[B46] de Cecco M. Criscione S. W. Peckham E. J. Hillenmeyer S. Hamm E. A. Manivannan J.. (2013). Genomes of replicatively senescent cells undergo global epigenetic changes leading to gene silencing and activation of transposable elements. Aging Cell 12, 247–256. 10.1111/acel.12047 23360310 PMC3618682

[B112] de Grey A. D. N. J. (2005). The plasma membrane redox system: a candidate source of aging-related oxidative stress. Age (Dordr) 27, 129–138. 10.1007/s11357-005-1630-1 23598619 PMC3458504

[B250] de Oliveira J. Hort M. A. Moreira E. L. G. Glaser V. Ribeiro-do-Valle R. M. Prediger R. D.. (2011). Positive correlation between elevated plasma cholesterol levels and cognitive impairments in LDL receptor knockout mice: Relevance of cortico-cerebral mitochondrial dysfunction and oxidative stress. Neuroscience 197, 99–106. 10.1016/j.neuroscience.2011.09.009 21945034

[B83] Deepashree S. Niveditha S. Shivanandappa T. Ramesh S. R. (2019). Oxidative stress resistance as a factor in aging: evidence from an extended longevity phenotype of *Drosophila melanogaster*. Biogerontology 20, 497–513. 10.1007/s10522-019-09812-7 31054025

[B84] Deschênes M. Chabot B. (2017). The emerging role of alternative splicing in senescence and aging. Aging Cell 16, 918–933. 10.1111/acel.12646 28703423 PMC5595669

[B86] Devaraj S. Jialal I. (2005). Failure of vitamin E in clinical trials: is gamma-tocopherol the answer? Nutr. Rev. 63, 290–293. 10.1111/j.1753-4887.2005.tb00143.x 16190316

[B87] Dhahbi J. M. Atamna H. Boffelli D. Magis W. Spindler S. R. Martin D. I. K. (2011). Deep sequencing reveals novel micrornas and regulation of microRNA expression during cell senescence. PLoS One 6:e20509. 10.1371/journal.pone.0020509 21637828 PMC3102725

[B91] Di Emidio G. Falone S. Vitti M. D’Alessandro A. M. Vento M. di Pietro C.. (2014). SIRT1 signalling protects mouse oocytes against oxidative stress and is deregulated during aging. Hum. Reprod. 29, 2006–2017. 10.1093/humrep/deu160 24963165

[B88] DiMauro S. Schon E. A. (2003). Mitochondrial respiratory-chain diseases. N. Engl. J. Med. 348, 2656–2668. 10.1056/NEJMra022567 12826641

[B89] Ding Y. Wang H. Niu J. Luo M. Gou Y. Miao L.. (2016). Induction of ROS overload by alantolactone prompts oxidative DNA damage and apoptosis in colorectal cancer cells. Int. J. Mol. Sci. 17:558. 10.3390/ijms17040558 27089328 PMC4849014

[B90] D’Orazio J. Jarrett S. Amaro-Ortiz A. Scott T. (2013). UV radiation and the skin. Int. J. Mol. Sci. 14, 12222–12248. 10.3390/ijms140612222 23749111 PMC3709783

[B92] Esworthy R. S. Aranda R. Martín M. G. Binder J. H. Doroshow S. W. Chu F.-F. (2001). Mice with combined disruption of *Gpx1* and *Gpx2* genes have colitis. Am. J. Physiol. Gastrointest. Liver Physiol. 281, G848–G855. 10.1152/ajpgi.2001.281.3.G848 11518697

[B93] Faggioli F. Wang T. Vijg J. Montagna C. (2012). Chromosome-specific accumulation of aneuploidy in the aging mouse brain. Hum. Mol. Genet. 21, 5246–5253. 10.1093/hmg/dds375 22962300 PMC3510757

[B94] Farris S. G. Abrantes A. M. (2020). Mental health benefits from lifestyle physical activity interventions: a systematic review. Bull. Menninger Clin. 84, 337–372. 10.1521/bumc.2020.84.4.337 33779237

[B95] Fernández-Agüera M. C. Gao L. González-Rodríguez P. Pintado C. O. Arias-Mayenco I. García-Flores P.. (2015). Oxygen sensing by arterial chemoreceptors depends on mitochondrial complex I signaling. Cell Metab. 22, 825–837. 10.1016/j.cmet.2015.09.004 26437605

[B96] Fernández-Ayala D. J. M. Jiménez-Gancedo S. Guerra I. Navas P. (2014). Invertebrate models for coenzyme q10 deficiency. Mol. Syndromol. 5, 170–179. 10.1016/j.ympev.2022.107526 25126050 PMC4112529

[B97] Fonseca L. J. S. D. Nunes-Souza V. Goulart M. O. F. Rabelo L. A. (2019). Oxidative stress in rheumatoid arthritis: what the future might hold regarding novel biomarkers and add-on therapies. Oxid. Med. Cell. Longev. 2019:7536805. 10.1155/2019/7536805 31934269 PMC6942903

[B98] Freund A. Orjalo A. V. Desprez P.-Y. Campisi J. (2010). Inflammatory networks during cellular senescence: causes and consequences. Trends Mol. Med. 16, 238–246. 10.1016/j.molmed.2010.03.003 20444648 PMC2879478

[B99] Freund A. Patil C. K. Campisi J. (2011). P38MAPK is a novel DNA damage response-independent regulator of the senescence-associated secretory phenotype. EMBO J. 30, 1536–1548. 10.1038/emboj.2011.69 21399611 PMC3102277

[B100] Fridovich I. (1998). Oxygen toxicity: a radical explanation. J. Exp. Biol. 201, 1203–1209. 10.1242/jeb.201.8.1203 9510531

[B101] Fujita K. Nishizawa H. Funahashi T. Shimomura I. Shimabukuro M. (2006). Systemic oxidative stress is associated with visceral fat accumulation and the metabolic syndrome. Circ. J. 70, 1437–1442. 10.1253/circj.70.1437 17062967

[B102] Fulco M. Sartorelli V. (2008). Comparing and contrasting the roles of AMPK and SIRT1 in metabolic tissues. Cell Cycle 7, 3669–3679. 10.4161/cc.7.23.7164 19029811 PMC2607479

[B103] Furukawa A. Tada-Oikawa S. Kawanishi S. Oikawa S. (2007). H_2_O_2_ accelerates cellular senescence by accumulation of acetylated p53 via decrease in the function of SIRT1 by NAD depletion. Cell. Physiol. Biochem. 20, 45–54. 10.1159/000104152 17595514

[B104] Galimov E. R. (2010). The role of p66shc in oxidative stress and apoptosis. Acta Naturae 2, 44–51. 10.32607/20758251-2010-2-4-44-51 22649663 PMC3347587

[B105] Gambino V. de Michele G. Venezia O. Migliaccio P. Dall’Olio V. Bernard L.. (2013). Oxidative stress activates a specific p53 transcriptional response that regulates cellular senescence and aging. Aging Cell 12, 435–445. 10.1111/acel.12060 23448364 PMC3709138

[B106] Gao X. Gào X. Zhang Y. Breitling L. P. Schöttker B. Brenner H. (2017). Associations of self-reported smoking, cotinine levels and epigenetic smoking indicators with oxidative stress among older adults: a population-based study. Eur. J. Epidemiol. 32, 443–456. 10.1007/s10654-017-0248-9 28434075

[B107] García-Sánchez A. Miranda-Díaz A. G. Cardona-Muñoz E. G. (2020). The role of oxidative stress in physiopathology and pharmacological treatment with pro- and antioxidant properties in chronic diseases. Oxid. Med. Cell. Longev. 2020:2082145. 10.1155/2020/2082145 32774665 PMC7396016

[B108] Ghezzi P. Jaquet V. Marcucci F. Schmidt H. H. H. W. (2017). The oxidative stress theory of disease: levels of evidence and epistemological aspects. Br. J. Pharmacol. 174, 1784–1796. 10.1111/bph.13544 27425643 PMC5446567

[B109] Gladyshev V. N. (2014). The free radical theory of aging is dead. Long live the damage theory!. Antioxid. Redox Signal. 20, 727–731. 10.1089/ars.2013.5228 24159899 PMC3901353

[B110] Goo H.-G. Jung M. K. Han S. S. Rhim H. Kang S. (2013). HtrA2/Omi deficiency causes damage and mutation of mitochondrial DNA. Biochim. Biophys. Acta 1833, 1866–1875. 10.1016/j.bbamcr.2013.03.016 23542127

[B111] Grabowska W. Sikora E. Bielak-Zmijewska A. (2017). Sirtuins, a promising target in slowing down the ageing process. Biogerontology 18, 447–476. 10.1007/s10522-017-9685-9 28258519 PMC5514220

[B113] Guarente L. Mostoslavsky R. Kazantsev A. Eds. (2018). Introductory Review on Sirtuins in Biology, Aging and Disease. Elsevier Inc. Imprint Academic Press. 10.1016/C2016-0-04790-3

[B114] Gu Y. Han J. Jiang C. Zhang Y. (2020). Biomarkers, oxidative stress and autophagy in skin aging. Ageing Res. Rev. 59:101036. 10.1016/j.arr.2020.101036 32105850

[B115] Hackl M. Brunner S. Fortschegger K. Schreiner C. Micutkova L. Mück C.. (2010). miR-17, miR-19b, miR-20a and miR-106a are down-regulated in human aging. Aging Cell 9, 291–296. 10.1111/j.1474-9726.2010.00549.x 20089119 PMC2848978

[B116] Hajam Y. A. Rani R. Ganie S. Y. Sheikh T. A. Javaid D. Qadri S. S.. (2022). Oxidative stress in human pathology and aging: molecular mechanisms and perspectives. Cells 11:552. 10.3390/cells11030552 35159361 PMC8833991

[B117] Harman D. (1956). Aging: a theory based on free radical and radiation chemistry. J. Gerontol. 11, 298–300. 10.1093/geronj/11.3.298 13332224

[B118] Harrison D. E. Strong R. Sharp Z. D. Nelson J. F. Astle C. M. Flurkey K.. (2009). Rapamycin fed late in life extends lifespan in genetically heterogeneous mice. Nature 460, 392–395. 10.1038/nature08221 19587680 PMC2786175

[B119] Hayashi Y. Yoshida M. Yamato M. Ide T. Wu Z. Ochi-Shindou M.. (2008). Reverse of age-dependent memory impairment and mitochondrial DNA damage in microglia by an overexpression of human mitochondrial transcription factor A in mice. J. Neurosci. 28, 8624–8634. 10.1523/JNEUROSCI.1957-08.2008 18716221 PMC6671051

[B120] Hayflick L. (1965). The limited *in vitro* lifetime of human diploid cell strains. Exp. Cell Res. 37, 614–636. 10.1016/0014-4827(65)90211-9 14315085

[B121] Herbig U. Ferreira M. Condel L. Carey D. Sedivy J. M. (2006). Cellular senescence in aging primates. Science 311:1257. 10.1126/science.1122446 16456035

[B122] Hickson L. J. Langhi Prata L. G. P. Bobart S. A. Evans T. K. Giorgadze N. Hashmi S. K.. (2019). Senolytics decrease senescent cells in humans: preliminary report from a clinical trial of Dasatinib plus Quercetin in individuals with diabetic kidney disease. EBioMedicine 47, 446–456. 10.1016/j.ebiom.2019.08.069 31542391 PMC6796530

[B123] Hiona A. Sanz A. Kujoth G. C. Pamplona R. Seo A. Y. Hofer T.. (2010). Mitochondrial DNA mutations induce mitochondrial dysfunction, apoptosis and sarcopenia in skeletal muscle of mitochondrial DNA mutator mice. PLoS One 5:e11468. 10.1371/journal.pone.0011468 20628647 PMC2898813

[B124] Hirst J. King M. S. Pryde K. R. (2008). The production of reactive oxygen species by complex I. Biochem. Soc. Trans. 36, 976–980. 10.1042/BST0360976 18793173

[B126] Ho Y.-S. Magnenat J.-L. Bronson R. T. Cao J. Gargano M. Sugawara M.. (1997). Mice deficient in cellular glutathione peroxidase develop normally and show no increased sensitivity to hyperoxia. J. Biol. Chem. 272, 16644–16651. 10.1074/jbc.272.26.16644 9195979

[B127] Ho Y.-S. Xiong Y. Ma W. Spector A. Ho D. S. (2004). Mice lacking catalase develop normally but show differential sensitivity to oxidant tissue injury. J. Biol. Chem. 279, 32804–32812. 10.1074/jbc.M404800200 15178682

[B125] Howitz K. T. Bitterman K. J. Cohen H. Y. Lamming D. W. Lavu S. Wood J. G.. (2003). Small molecule activators of sirtuins extend *Saccharomyces cerevisiae* lifespan. Nature 425, 191–196. 10.1038/nature01960 12939617

[B128] Hubackova S. Krejcikova K. Bartek J. Hodny Z. (2012). IL1-and TGFβ-Nox4 signaling, oxidative stress and DNA damage response are shared features of replicative, oncogene-induced and drug-induced paracrine “Bystander senescence”. Aging (Albany NY) 4, 932–951. 10.18632/aging.100520 23385065 PMC3615160

[B129] Hussein A. M. Hasan S. (2010). Cadmium affects viability of bone marrow mesenchymal stem cells through membrane impairment, intracellular calcium elevation and DNA breakage. Indian J. Med. Sci. 64, 177–186. 10.4103/0019-5359.97357 22718012

[B130] Hyun B.-H. Emerson S. S. Jo D.-G. Mattson M. P. de Cabo R. (2006). Calorie restriction up-regulates the plasma membrane redox system in brain cells and suppresses oxidative stress during aging. Proc. Natl. Acad. Sci. U S A 103, 19908–19912. 10.1073/pnas.0608008103 17167053 PMC1750890

[B131] Hyun D.-H. Hernandez J. O. Mattson M. P. de Cabo R. (2006). The plasma membrane redox system in aging. Ageing Res. Rev. 5, 209–220. 10.1016/j.arr.2006.03.005 16697277

[B132] Ibáñez-Ventoso C. Yang M. Guo S. Robins H. Padgett R. W. Driscoll M. (2006). Modulated microRNA expression during adult lifespan in *Caenorhabditis elegans*. Aging Cell 5, 235–246. 10.1111/j.1474-9726.2006.00210.x 16842496

[B133] Ichimura H. Parthasarathi K. Quadri S. Issekutz A. C. Bhattacharya J. (2003). Mechano-oxidative coupling by mitochondria induces proinflammatory responses in lung venular capillaries. J. Clin. Invest. 111, 691–699. 10.1172/JCI17271 12618523 PMC151903

[B134] Inukai S. de Lencastre A. Turner M. Slack F. (2012). Novel microRNAs differentially expressed during aging in the mouse brain. PLoS One 7:e40028. 10.1371/journal.pone.0040028 22844398 PMC3402511

[B135] Jacinto E. Loewith R. Schmidt A. Lin S. Rüegg M. A. Hall A.. (2004). Mammalian TOR complex 2 controls the actin cytoskeleton and is rapamycin insensitive. Nat. Cell Biol. 6, 1122–1128. 10.1038/ncb1183 15467718

[B136] Janssen B. G. Munters E. Pieters N. Smeets K. Cox B. Cuypers A.. (2012). Placental mitochondrial DNA content and particulate air pollution during *in utero* life. Environ. Health Perspect. 120, 1346–1352. 10.1289/ehp.1104458 22626541 PMC3440109

[B137] Jazbec K. Jež M. Justin M. Rožman P. (2019). Molecular mechanisms of stem cell aging. Sloven. Vet. Res. 56, 5–12. 10.26873/SVR-545-2018

[B138] Jeyapalan J. C. Ferreira M. Sedivy J. M. Herbig U. (2007). Accumulation of senescent cells in mitotic tissue of aging primates. Mech. Ageing Dev. 128, 36–44. 10.1016/j.mad.2006.11.008 17116315 PMC3654105

[B140] Jiang H. Schiffer E. Song Z. Wang J. Zürbig P. Thedieck K.. (2008). Proteins induced by telomere dysfunction and DNA damage represent biomarkers of human aging and disease. Proc. Natl. Acad. Sci. U S A 105, 11299–11304. 10.1073/pnas.0801457105 18695223 PMC2516278

[B139] Jiang G. Xu L. Zhang B. Wu L. (2011). Effects of cadmium on proliferation and self-renewal activity of prostate stem/progenitor cells. Environ. Toxicol. Pharmacol. 32, 275–284. 10.1016/j.etap.2011.05.015 21843809

[B141] Johnson S. C. Rabinovitch P. S. Kaeberlein M. (2013). MTOR is a key modulator of ageing and age-related disease. Nature 493, 338–345. 10.1038/nature11861 23325216 PMC3687363

[B142] Juan C. A. de la Lastra J. M. P. Plou F. J. Pérez-Lebeña E. (2021). The chemistry of reactive oxygen species (Ros) revisited: outlining their role in biological macromolecules (DNA, lipids and proteins) and induced pathologies. Int. J. Mol. Sci. 22. 10.3390/ijms22094642 33924958 PMC8125527

[B143] Jungert A. Neuhäuser-Berthold M. (2018). Cross-sectional and longitudinal associations between serum 25-hydroxyvitamin D and anti-oxidative status in older adults. Exp. Gerontol. 110, 291–297. 10.1016/j.exger.2018.06.024 29953952

[B144] Jurk D. Wilson C. Passos J. F. Oakley F. Correia-Melo C. Greaves L.. (2014). Chronic inflammation induces telomere dysfunction and accelerates ageing in mice. Nat. Commun. 2:4172. 10.1038/ncomms5172 24960204 PMC4090717

[B145] Kaitsuka T. Matsushita M. Matsushita N. (2021). Regulation of hypoxic signaling and oxidative stress via the microrna-sirt2 axis and its relationship with aging-related diseases. Cells 10:3316. 10.3390/cells10123316 34943825 PMC8699081

[B146] Kandola K. Bowman A. Birch-Machin M. A. (2015). Oxidative stress - a key emerging impact factor in health, ageing, lifestyle and aesthetics. Int. J. Cosmet. Sci. 37, 1–8. 10.1111/ics.12287 26574299

[B147] Kanfi Y. Naiman S. Amir G. Peshti V. Zinman G. Nahum L.. (2012). The sirtuin SIRT6 regulates lifespan in male mice. Nature 483, 218–221. 10.1038/nature10815 22367546

[B148] Kao C.-L. Chen L.-K. Chang Y.-L. Yung M.-C. Hsu C.-C. Chen Y.-C.. (2010). Resveratrol protects human endothelium from H_2_O_2_-induced oxidative stress and senescence via SirT1 activation. J. Atheroscler. Thromb. 17, 970–979. 10.5551/jat.4333 20644332

[B149] Karol M. H. (2009). How environmental agents influence the aging process. Biomol. Ther. 17, 113–124. 10.4062/biomolther.2009.17.2.113

[B150] Kaufman B. A. Durisic N. Mativetsky J. M. Costantino S. Hancock M. A. Grutter P.. (2007). The mitochondrial transcription factor TFAM coordinates the assembly of multiple DNA molecules into nucleoid-like structures. Mol. Biol. Cell 18, 3225–3236. 10.1091/mbc.e07-05-0404 17581862 PMC1951767

[B151] Keaney M. Gems D. (2003). No increase in lifespan in Caenorhabditis elegans upon treatment with the superoxide dismutase mimetic EUK-8. Free Radic. Biol. Med. 34, 277–282. 10.1016/s0891-5849(02)01290-x 12521609

[B152] Keaney M. Matthijssens F. Sharpe M. Vanfleteren J. Gems D. (2004). Superoxide dismutase mimetics elevate superoxide dismutase activity *in vivo* but do not retard aging in the nematode *Caenorhabditis elegans*. Free Radic. Biol. Med. 37, 239–250. 10.1016/j.freeradbiomed.2004.04.005 15203195

[B153] Khapre R. V. Kondratova A. A. Patel S. Dubrovsky Y. Wrobel M. Antoch M. P.. (2014). BMAL1-dependent regulation of the mTOR signaling pathway delays aging. Aging (Albany NY) 6, 48–57. 10.18632/aging.100633 24481314 PMC3927809

[B154] Kim J.-H. Na H.-J. Kim C.-K. Kim J.-Y. Ha K.-S. Lee H.. (2008). The non-provitamin A carotenoid, lutein, inhibits NF-κB-dependent gene expression through redox-based regulation of the phosphatidylinositol 3-kinase/PTEN/Akt and NF-κB-inducing kinase pathways: role of H_2_O_2_ in NF-κB activation. Free Radic. Biol. Med. 45, 885–896. 10.1016/j.freeradbiomed.2008.06.019 18620044

[B156] Kirkwood T. B. L. (2005a). “The biological science of human ageing,” in The Cambridge Handbook of Age and Ageing ed M. L. Johnson (Cambridge: Cambridge University Press), 72–81. Available online at: https://www.cambridge.org/core/books/cambridge-handbook-of-age-and-ageing/DBC1E77201A4414EF67DA2DF6D2D4667

[B155] Kirkwood T. B. L. (2005b). Understanding the odd science of aging. Cell 120, 437–447. 10.1016/j.cell.2005.01.027 15734677

[B157] Kirkwood T. B. L. Austad S. N. (2000). Why do we age. Nature 408, 233–238. 10.1038/35041682 11089980

[B158] Kiss T. Giles C. B. Tarantini S. Yabluchanskiy A. Balasubramanian P. Gautam T.. (2019). Nicotinamide mononucleotide (NMN) supplementation promotes anti-aging miRNA expression profile in the aorta of aged mice, predicting epigenetic rejuvenation and anti-atherogenic effects. GeroScience 41, 419–439. 10.1007/s11357-019-00095-x 31463647 PMC6815288

[B159] Koc A. Gasch A. P. Rutherford J. C. Kim H.-Y. Gladyshev V. N. (2004). Methionine sulfoxide reductase regulation of yeast lifespan reveals reactive oxygen species-dependent and -independent components of aging. Proc. Natl. Acad. Sci. U S A 101, 7999–8004. 10.1073/pnas.0307929101 15141092 PMC419546

[B160] Kothapalli C. R. (2021). Differential impact of heavy metals on neurotoxicity during development and in aging central nervous system. Curr. Opin. Toxicol. 26, 33–38. 10.1016/j.cotox.2021.04.003

[B161] Kraaij M. D. Savage N. D. L. van der Kooij S. W. Koekkoek K. Wang J. van den Berg J. M.. (2010). Induction of regulatory T cells by macrophages is dependent on production of reactive oxygen species. Proc. Natl. Acad. Sci. U S A 107, 17686–17691. 10.1073/pnas.1012016107 20861446 PMC2955141

[B162] Krishnamurthy J. Ramsey M. R. Ligon K. L. Torrice C. Koh A. Bonner-Weir S.. (2006). p16^INK4a^ induces an age-dependent decline in islet regenerative potential. Nature 443, 453–457. 10.1038/nature05092 16957737

[B163] Krutmann J. Liu W. Li L. Pan X. Crawford M. Sore G.. (2014). Pollution and skin: from epidemiological and mechanistic studies to clinical implications. J. Dermatol. Sci. 76, 163–168. 10.1016/j.jdermsci.2014.08.008 25278222

[B164] Krutmann J. Schroeder P. (2009). Role of mitochondria in photoaging of human skin: the defective powerhouse model. J. Investig. Dermatol. Symp. Proc. 14, 44–49. 10.1038/jidsymp.2009.1 19675552

[B165] Kuilman T. Michaloglou C. Vredeveld L. C. W. Douma S. van Doorn R. Desmet C. J.. (2008). Oncogene-induced senescence relayed by an interleukin-dependent inflammatory network. Cell 133, 1019–1031. 10.1016/j.cell.2008.03.039 18555778

[B166] Kujoth C. C. Hiona A. Pugh T. D. Someya S. Panzer K. Wohlgemuth S. E.. (2005). Medicine: mitochondrial DNA mutations, oxidative stress and apoptosis in mammalian aging. Science 309, 481–484. 10.1126/science.1112125 16020738

[B167] Kulkarni S. S. Cantó C. (2015). The molecular targets of resveratrol. Biochim. Biophys. Acta 1852, 1114–1123. 10.1016/j.bbadis.2014.10.005 25315298

[B168] Kurutas E. B. (2016). The importance of antioxidants which play the role in cellular response against oxidative/nitrosative stress: Current state. Nutr. J. 15:71. 10.1186/s12937-016-0186-5 27456681 PMC4960740

[B169] Laberge R.-M. Sun Y. Orjalo A. V. Patil C. K. Freund A. Zhou L.. (2015). MTOR regulates the pro-tumorigenic senescence-associated secretory phenotype by promoting IL1A translation. Nat. Cell Biol. 17, 1049–1061. 10.1038/ncb3195 26147250 PMC4691706

[B170] Lagnado A. Leslie J. Ruchaud-Sparagano M.-H. Victorelli S. Hirsova P. Ogrodnik M.. (2021). Neutrophils induce paracrine telomere dysfunction and senescence in ROS-dependent manner. EMBO J. 40:e106048. 10.15252/embj.2020106048 33764576 PMC8090854

[B171] Lane R. K. Hilsabeck T. Rea S. L. (2015). The role of mitochondrial dysfunction in age-related diseases. Biochim. Biophys. Acta 1847, 1387–1400. 10.1016/j.bbabio.2015.05.021 26050974 PMC10481969

[B172] LaPak K. M. Burd C. E. (2014). The molecular balancing act of p16^ink4a^ in cancer and aging. Mol. Cancer Res. 12, 167–183. 10.1158/1541-7786.MCR-13-0350 24136988 PMC3944093

[B173] Latimer J. A. Lloyd J. J. Diffey B. L. Matts P. J. Birch-Machin M. A. (2015). Determination of the action spectrum of UVR-induced mitochondrial DNA damage in human skin cells. J. Invest. Dermatol. 135, 2512–2518. 10.1038/jid.2015.194 26030182

[B174] Lee C. Longo V. (2016). Dietary restriction with and without caloric restriction for healthy aging. F1000Res. 5: .F1000 Faculty Rev-117. 10.12688/f1000research.7136.1 26918181 PMC4755412

[B175] Lee K.-S. Shin S. Cho E. Im W. K. Jeon S. H. Kim Y.. (2019). nc886, a non-coding RNA, inhibits UVB-induced MMP-9 and COX-2 expression via the PKR pathway in human keratinocytes. Biochem. Biophys. Res. Commun. 512, 647–652. 10.1016/j.bbrc.2019.01.068 30685091

[B177] Lee S. Tak E. Lee J. Rashid M. Murphy M. P. Ha J.. (2011). Mitochondrial H_2_O_2_ generated from electron transport chain complex i stimulates muscle differentiation. Cell Res. 21, 817–834. 10.1038/cr.2011.55 21445095 PMC3203677

[B176] Lee S.-R. Yang K.-S. Kwon J. Lee C. Jeong W. Rhee S. G. (2002). Reversible inactivation of the tumor suppressor PTEN by H_2_O_2_. J. Biol. Chem. 277, 20336–20342. 10.1074/jbc.M111899200 11916965

[B178] Lehrbach N. J. Castro C. Murfitt K. J. Abreu-Goodger C. Griffin J. L. Miska E. A. (2012). Post-developmental microRNA expression is required for normal physiology and regulates aging in parallel to insulin/IGF-1 signaling in C. elegans. RNA 18, 2220–2235. 10.1261/rna.035402.112 23097426 PMC3504673

[B179] Leontieva O. V. Paszkiewicz G. M. Blagosklonny M. V. (2012). Mechanistic or mammalian target of rapamycin (mTOR) may determine robustness in young male mice at the cost of accelerated aging. Aging (Albany NY) 4, 899–916. 10.18632/aging.100528 23443503 PMC3615157

[B180] Lesina M. Wörmann S. M. Morton J. Diakopoulos K. N. Korneeva O. Wimmer M.. (2016). RelA regulates CXCL1/CXCR2-dependent oncogene-induced senescence in murine Kras-driven pancreatic carcinogenesis. J. Clin. Invest. 126, 2919–2932. 10.1172/JCI86477 27454298 PMC4966329

[B181] Lewinska A. Adamczyk-Grochala J. Kwasniewicz E. Deregowska A. Semik E. Zabek T.. (2018). Reduced levels of methyltransferase DNMT2 sensitize human fibroblasts to oxidative stress and DNA damage that is accompanied by changes in proliferation-related miRNA expression. Redox Biol. 14, 20–34. 10.1016/j.redox.2017.08.012 28843151 PMC5568885

[B182] Liang X. Zhang D. Liu W. Yan Y. Zhou F. Wu W.. (2017). Reactive oxygen species trigger NF-κB-mediated NLRP3 inflammasome activation induced by zinc oxide nanoparticles in A549 cells. Toxicol. Ind. Health 33, 737–745. 10.1177/0748233717712409 28870124

[B183] Liao C.-Y. Kennedy B. K. (2016). SIRT6, oxidative stress and aging. Cell Res. 26, 143–144. 10.1038/cr.2016.8 26780861 PMC4746614

[B186] Li H. Chen F.-J. Yang W.-L. Qiao H.-Z. Zhang S.-J. (2021). Quercetin improves cognitive disorder in aging mice by inhibiting NLRP3 inflammasome activation. Food Funct. 12, 717–725. 10.1039/d0fo01900c 33338087

[B184] Li F.-H. Sun L. Zhu M. Li T. Gao H.-E. Wu D.-S.. (2018). Beneficial alterations in body composition, physical performance, oxidative stress, inflammatory markers and adipocytokines induced by long-term high-intensity interval training in an aged rat model. Exp. Gerontol. 113, 150–162. 10.1016/j.exger.2018.10.006 30308288

[B196] Li Y. Yao J. Han C. Yang J. Chaudhry M. T. Wang S.. (2016). Quercetin, inflammation and immunity. Nutrients 8:167. 10.3390/nu8030167 26999194 PMC4808895

[B187] Li M. Zhao L. Liu J. Liu A. Jia C. Ma D.. (2010). Multi-mechanisms are involved in reactive oxygen species regulation of mTORC1 signaling. Cell Signal. 22, 1469–1476. 10.1016/j.cellsig.2010.05.015 20639120

[B185] Liguori I. Russo G. Curcio F. Bulli G. Aran L. Della-Morte D.. (2018). Oxidative stress, aging and diseases. Clin. Interv. Aging 13, 757–772. 10.2147/CIA.S158513 29731617 PMC5927356

[B188] Lin C.-H. Lin C.-C. Ting W.-J. Pai P.-Y. Kuo C.-H. Ho T.-J.. (2014). Resveratrol enhanced FOXO3 phosphorylation via synergetic activation of SIRT1 and PI3K/Akt signaling to improve the effects of exercise in elderly rat hearts. Age (Dordr) 36:9705. 10.1007/s11357-014-9705-5 25158994 PMC4453936

[B190] Lin X.-X. Sen I. Janssens G. E. Zhou X. Fonslow B. R. Edgar D.. (2018). DAF-16/FOXO and HLH-30/TFEB function as combinatorial transcription factors to promote stress resistance and longevity. Nat. Commun. 9:4400. 10.1038/s41467-018-06624-0 30353013 PMC6199276

[B191] Lin Z. Yang H. Tan C. Li J. Liu Z. Quan Q.. (2013). USP10 Antagonizes c-Myc transcriptional activation through SIRT6 stabilization to suppress tumor formation. Cell Rep. 5, 1639–1649. 10.1016/j.celrep.2013.11.029 24332849 PMC4007576

[B189] Linnane A. W. Ozawa T. Marzuki S. Tanaka M. (1989). Mitochondrial DNA mutations as an important contributor to ageing and degenerative diseases. Lancet 333, 642–645. 10.1016/s0140-6736(89)92145-4 2564461

[B192] Littler J. S. (1978). Reaction mechanisms in organic chemistry. Endeavour 2.

[B193] Liu J. Chen S. Biswas S. Nagrani N. Chu Y. Chakrabarti S.. (2020). Glucose-induced oxidative stress and accelerated aging in endothelial cells are mediated by the depletion of mitochondrial SIRTs. Physiol. Rep. 8:e14331. 10.14814/phy2.14331 32026628 PMC7002531

[B195] Liu Z. Jiang C. Zhang J. Liu B. Du Q. (2016). Resveratrol inhibits inflammation and ameliorates insulin resistant endothelial dysfunction via regulation of AMP-activated protein kinase and sirtuin 1 activities. J. Diabetes 8, 324–335. 10.1111/1753-0407.12296 25850408

[B194] Liu J. Khalil R. A. (2017). Matrix metalloproteinase inhibitors as investigational and therapeutic tools in unrestrained tissue remodeling and pathological disorders. Prog. Mol. Biol. Transl. Sci. 148, 355–420. 10.1016/bs.pmbts.2017.04.003 28662828 PMC5548434

[B197] Longo V. D. Mitteldorf J. Skulachev V. P. (2005). Opinion: programmed and altruistic ageing. Nat. Rev. Genet. 6, 866–872. 10.1038/nrg1706 16304601

[B198] López-Otín C. Blasco M. A. Partridge L. Serrano M. Kroemer G. (2013). The hallmarks of aging. Cell 153:1194. 10.1016/j.cell.2013.05.039 23746838 PMC3836174

[B199] Ludlow A. T. Spangenburg E. E. Chin E. R. Cheng W.-H. Roth S. M. (2014). Telomeres shorten in response to oxidative stress in mouse skeletal muscle fibers. J. Gerontol. A Biol. Sci. Med. Sci. 69, 821–830. 10.1093/gerona/glt211 24418792 PMC4111633

[B200] Luo J. Mills K. le Cessie S. Noordam R. van Heemst D. (2020). Ageing, age-related diseases and oxidative stress: What to do next. Ageing Res. Rev. 57:100982. 10.1016/j.arr.2019.100982 31733333

[B201] Madani A. Y. Majeed Y. Abdesselem H. B. Agha M. V. Vakayil M. al Sukhun N. K.. (2021). Signal transducer and activator of transcription 3 (Stat3) suppresses stat1/interferon signaling pathway and inflammation in senescent preadipocytes. Antioxidants (Basel) 10:334. 10.3390/antiox10020334 33672392 PMC7927067

[B202] Magalhães S. Goodfellow B. J. Nunes A. (2018). Aging and proteins: what does proteostasis have to do with age. Curr. Mol. Med. 18, 178–189. 10.2174/1566524018666180907162955 30198430

[B203] Magenta A. Cencioni C. Fasanaro P. Zaccagnini G. Greco S. Sarra-Ferraris G.. (2011). MiR-200c is upregulated by oxidative stress and induces endothelial cell apoptosis and senescence via ZEB1 inhibition. Cell Death Diff. 18, 1628–1639. 10.1038/cdd.2011.42 21527937 PMC3172120

[B204] Ma K. Chen G. Li W. Kepp O. Zhu Y. Chen Q. (2020). Mitophagy, mitochondrial homeostasis and cell fate. Front. Cell Dev. Biol. 8:467. 10.3389/fcell.2020.00467 32671064 PMC7326955

[B205] Mamoshina P. Kochetov K. Cortese F. Kovalchuk A. Aliper A. Putin E.. (2019). Blood biochemistry analysis to detect smoking status and quantify accelerated aging in smokers. Sci. Rep. 9:142. 10.1038/s41598-018-35704-w 30644411 PMC6333803

[B206] Mannick J. B. del Giudice G. Lattanzi M. Valiante N. M. Praestgaard J. Huang B.. (2014). mTOR inhibition improves immune function in the elderly. Sci. Transl. Med. 6:268ra179. 10.1126/scitranslmed.3009892 25540326

[B207] Marin C. Delgado-Lista J. Ramirez R. Carracedo J. Caballero J. Perez-Martinez P.. (2012). Mediterranean diet reduces senescence-associated stress in endothelial cells. Age (Dordr) 34, 1309–1316. 10.1007/s11357-011-9305-6 21894446 PMC3528364

[B208] Markevich N. I. Markevich L. N. Hoek J. B. (2020). Computational modeling analysis of generation of reactive oxygen species by mitochondrial assembled and disintegrated complex II. Front. Physiol. 11:557721. 10.3389/fphys.2020.557721 33178032 PMC7596731

[B209] Martens D. S. Cox B. Janssen B. G. Clemente D. B. P. Gasparrini A. Vanpoucke C.. (2017). Prenatal air pollution and newborns’ predisposition to accelerated biological aging. JAMA Pediatr. 171, 1160–1167. 10.1001/jamapediatrics.2017.3024 29049509 PMC6233867

[B210] Martens D. S. Nawrot T. S. (2018). Ageing at the level of telomeres in association to residential landscape and air pollution at home and work: a review of the current evidence. Toxicol. Lett. 298, 42–52. 10.1016/j.toxlet.2018.06.1213 29944903

[B211] Martínez-Reyes I. Chandel N. S. (2020). Mitochondrial TCA cycle metabolites control physiology and disease. Nat. Commun. 11:102. 10.1038/s41467-019-13668-3 31900386 PMC6941980

[B212] Matsuda M. Shimomura I. (2013). Increased oxidative stress in obesity: Implications for metabolic syndrome, diabetes, hypertension, dyslipidemia, atherosclerosis and cancer. Obes. Res. Clin. Pract. 7, e330–e341. 10.1016/j.orcp.2013.05.004 24455761

[B213] Mattison J. A. Wang M. Bernier M. Zhang J. Park S.-S. Maudsley S.. (2014). Resveratrol prevents high fat/sucrose diet-induced central arterial wall inflammation and stiffening in nonhuman primates. Cell Metab. 20, 183–190. 10.1016/j.cmet.2014.04.018 24882067 PMC4254394

[B214] Maurya P. K. Noto C. Rizzo L. B. Rios A. C. Nunes S. O. V. Barbosa D. S.. (2016). The role of oxidative and nitrosative stress in accelerated aging and major depressive disorder. Prog. NeuroPsychopharmacol. Biol. Psychiatry 65, 134–144. 10.1016/j.pnpbp.2015.08.016 26348786

[B215] Maynou L. Hernández-pizarro H. M. Rodríguez M. E. (2021). The association of physical (on)activity with mental health. differences between elder and younger populations: a systematic literature review. Int. J. Environ. Res. Public Health 18:4771. 10.3390/ijerph18094771 33947122 PMC8124550

[B216] McCarthy D. A. Clark R. R. Bartling T. R. Trebak M. Melendez J. A. (2013). Redox control of the senescence regulator interleukin-1α and the secretory phenotype. J. Biol. Chem. 288, 32149–32159. 10.1074/jbc.M113.493841 24062309 PMC3820855

[B217] McConnell B. B. Starborg M. Brookes S. Peters G. (1998). Inhibitors of cyclin-dependent kinases induce features of replicative senescence in early passage human diploid fibroblasts. Curr. Biol. 8, 351–354. 10.1016/s0960-9822(98)70137-x 9512419

[B218] McCord J. M. (2000). The evolution of free radicals and oxidative stress. Am. J. Med. 108, 652–659. 10.1016/s0002-9343(00)00412-5 10856414

[B219] McCord J. M. Fridovich I. (1969). Superoxide dismutase. an enzymic function for erythrocuprein (hemocuprein). J. Biol. Chem. 244, 6049–6055. 5389100

[B220] Meehan M. Penckofer S. (2014). The role of vitamin D in the aging adult. J. Aging Gerontol. 2, 60–71. 10.12974/2309-6128.2014.02.02.1 25893188 PMC4399494

[B221] Meo S. D. Venditti P. (2001). Mitochondria in exercise-induced oxidative stress. Biol. Signals Recept. 10, 125–140. 10.1159/000046880 11223645

[B222] Miller R. A. Buehner G. Chang Y. Harper J. M. Sigler R. Smith-Wheelock M. (2005). Methionine-deficient diet extends mouse lifespan, slows immune and lens aging, alters glucose, T4, IGF-I and insulin levels and increases hepatocyte MIF levels and stress resistance. Aging Cell 4, 119–125. 10.1111/j.1474-9726.2005.00152.x 15924568 PMC7159399

[B223] Miller R. A. Harrison D. E. Astle C. M. Baur J. A. Boyd A. R. de Cabo R.. (2011). Rapamycin, but not resveratrol or simvastatin, extends life span of genetically heterogeneous mice. J. Gerontol. A Biol. Sci. Med. Sci. 66, 191–201. 10.1093/gerona/glq178 20974732 PMC3021372

[B224] Min L. Ji Y. Bakiri L. Qiu Z. Cen J. Chen X.. (2012). Liver cancer initiation is controlled by AP-1 through SIRT6-dependent inhibition of survivin. Nat. Cell Biol. 14, 1203–1211. 10.1038/ncb2590 23041974

[B225] Min S. K. Ki U. L. (2005). Role of hypothalamic 5′-AMP-activated protein kinase in the regulation of food intake and energy homeostasis. J. Mol. Med. 83, 514–520. 10.1007/s00109-005-0659-z 15806319

[B226] Mittal M. Siddiqui M. R. Tran K. Reddy S. P. Malik A. B. (2014). Reactive oxygen species in inflammation and tissue injury. Antioxid. Redox Signal. 20, 1126–1167. 10.1089/ars.2012.5149 23991888 PMC3929010

[B227] Mockett R. J. Bayne A.-C. V. Kwong L. K. Orr W. C. Sohal R. S. (2003). Ectopic expression of catalase in *Drosophila* mitochondria increases stress resistance but not longevity. Free Radic. Biol. Med. 34, 207–217. 10.1016/s0891-5849(02)01190-5 12521602

[B228] Mockett R. J. Sohal R. S. Orr W. C. (1999). Overexpression of glutathione reductase extends survival in transgenic *Drosophila melanogaster* under hyperoxia but not normoxia. FASEB J. 13, 1733–1742. 10.1096/fasebj.13.13.1733 10506576

[B229] Möller M. N. Rios N. Trujillo M. Radi R. Denicola A. Alvarez B. (2019). Detection and quantification of nitric oxide-derived oxidants in biological systems. J. Biol. Chem. 294, 14776–14802. 10.1074/jbc.REV119.006136 31409645 PMC6779446

[B230] Mostoslavsky R. Chua K. F. Lombard D. B. Pang W. W. Fischer M. R. Gellon L.. (2006). Genomic instability and aging-like phenotype in the absence of mammalian SIRT6. Cell 124, 315–329. 10.1016/j.cell.2005.11.044 16439206

[B231] Mrowicka M. Mrowicki J. Kucharska E. Smigielska B. Szaflik J. P. Szaflik J.. (2021). The role of oxidative stress and the importance of mirnas as potential biomarkers in the development of age-related macular degeneration. Processes 9:1328. 10.3390/pr9081328

[B232] Mueller M. M. Castells-Roca L. Babu V. Ermolaeva M. A. Müller R.-U. Frommolt P.. (2014). DAF-16/FOXO and EGL-27/GATA promote developmental growth in response to persistent somatic DNA damage. Nat. Cell Biol. 16, 1168–1179. 10.1038/ncb3071 25419847 PMC4250074

[B233] Muñoz-Espín D. Cañamero M. Maraver A. Gómez-López G. Contreras J. Murillo-Cuesta S.. (2013). Programmed cell senescence during mammalian embryonic development. Cell 155, 1104–1118. 10.1016/j.cell.2013.10.019 24238962

[B234] Münscher C. Müller-höcker J. Kadenbach B. (1993). Human aging is associated with various point mutations in tRNA genes of mitochondrial DNA. Biol. Chem. Hoppe Seyler 374, 1099–1104. 10.1515/bchm3.1993.374.7-12.1099 8129854

[B235] Murase T. Haramizu S. Ota N. Hase T. (2009). Suppression of the aging-associated decline in physical performance by a combination of resveratrol intake and habitual exercise in senescence-accelerated mice. Biogerontology 10, 423–434. 10.1007/s10522-008-9177-z 18830683

[B236] Murphy M. P. (2009). How mitochondria produce reactive oxygen species. Biochem. J. 417, 1–13. 10.1042/BJ20081386 19061483 PMC2605959

[B237] Nacarelli T. Azar A. Sell C. (2016). Mitochondrial stress induces cellular senescence in an mTORC1-dependent manner. Free Radic. Biol. Med. 95, 133–154. 10.1016/j.freeradbiomed.2016.03.008 27016071

[B238] Naidoo K. Birch-Machin M. A. (2017). Oxidative stress and ageing: The influence of environmental pollution, sunlight and diet on skin. Cosmetics 4:4. 10.3390/cosmetics4010004

[B240] Nauseef W. M. (2008). Biological roles for the NOX family NADPH oxidases. J. Biol. Chem. 283, 16961–16965. 10.1074/jbc.R700045200 18420576 PMC2427363

[B241] Nelson G. Wordsworth J. Wang C. Jurk D. Lawless C. Martin-Ruiz C.. (2012). A senescent cell bystander effect: senescence-induced senescence. Aging Cell 11, 345–349. 10.1111/j.1474-9726.2012.00795.x 22321662 PMC3488292

[B243] Nicita-Mauro V. Basile G. Maltese G. Nicita-Mauro C. Gangemi S. Caruso C. (2008a). Smoking, health and ageing. Immun. Ageing 5. 10.1186/1742-4933-5-10 PMC2564903 18796145

[B242] Nicita-Mauro V. lo Balbo C. Mento A. Nicita-Mauro C. Maltese G. Basile G. (2008b). Smoking, aging and the centenarians. Exp. Gerontol. 43, 95–101. 10.1016/j.exger.2007.06.011 17686596

[B244] Nijhawan P. Arora S. Behl T. (2019). Intricate role of oxidative stress in the progression of obesity. Obes. Med. 15:100125. 10.1016/j.obmed.2019.100125

[B245] Nogueira V. Park Y. Chen C.-C. Xu P.-Z. Chen M.-L. Tonic I.. (2008). Akt determines replicative senescence and oxidative or oncogenic premature senescence and sensitizes cells to oxidative apoptosis. Cancer Cell 14, 458–470. 10.1016/j.ccr.2008.11.003 19061837 PMC3038665

[B246] Novais E. J. Tran V. A. Johnston S. N. Darris K. R. Roupas A. J. Sessions G. A.. (2021). Long-term treatment with senolytic drugs Dasatinib and Quercetin ameliorates age-dependent intervertebral disc degeneration in mice. Nat. Commun. 12:5213. 10.1038/s41467-021-25453-2 34480023 PMC8417260

[B247] Novakova Z. Hubackova S. Kosar M. Janderova-Rossmeislova L. Dobrovolna J. Vasicova P.. (2010). Cytokine expression and signaling in drug-induced cellular senescence. Oncogene 29, 273–284. 10.1038/onc.2009.318 19802007

[B248] Nsiah-Sefaa A. McKenzie M. (2016). Combined defects in oxidative phosphorylation and fatty acid β-oxidation in mitochondrial disease. Biosci. Rep. 36:e00313. 10.1042/BSR20150295 26839416 PMC4793296

[B249] Ohata H. Shiokawa D. Obata Y. Sato A. Sakai H. Fukami M.. (2019). NOX1-dependent mTORC1 activation via S100A9 oxidation in cancer stem-like cells leads to colon cancer progression. Cell Rep. 28, 1282–1295.e8. 10.1016/j.celrep.2019.06.085 31365870

[B251] Onyango I. G. Dennis J. Khan S. M. (2016). Mitochondrial dysfunction in Alzheimer’s disease and the rationale for bioenergetics based therapies. Aging Dis. 7, 201–214. 10.14336/AD.2015.1007 27114851 PMC4809610

[B252] Orjalo A. V. Bhaumik D. Gengler B. K. Scott G. K. Campisi J. (2009). Cell surface-bound IL-1α is an upstream regulator of the senescence-associated IL-6/IL-8 cytokine network. Proc. Natl. Acad. Sci. U S A 106, 17031–17036. 10.1073/pnas.0905299106 19805069 PMC2761322

[B253] Ota H. Akishita M. Eto M. Iijima K. Kaneki M. Ouchi Y. (2007). Sirt1 modulates premature senescence-like phenotype in human endothelial cells. J. Mol. Cell. Cardiol. 43, 571–579. 10.1016/j.yjmcc.2007.08.008 17916362

[B254] Ota H. Tokunaga E. Chang K. Hikasa M. Iijima K. Eto M.. (2006). Sirt1 inhibitor, Sirtinol, induces senescence-like growth arrest with attenuated Ras-MAPK signaling in human cancer cells. Oncogene 25, 176–185. 10.1038/sj.onc.1209049 16170353

[B255] Outeiro T. F. Kontopoulos E. Altmann S. M. Kufareva I. Strathearn K. E. Amore A. M.. (2007). Sirtuin 2 inhibitors rescue α-synuclein-mediated toxicity in models of Parkinson’s disease. Science 317, 516–519. 10.1126/science.1143780 17588900

[B256] Padmavathi P. Raghu P. S. Reddy V. D. Bulle S. Marthadu S. B. Maturu P.. (2018). Chronic cigarette smoking-induced oxidative/nitrosative stress in human erythrocytes and platelets. Mol. Cell. Toxicol. 14, 27–34. 10.1007/s13273-018-0004-6

[B257] Pal S. Tyler J. K. (2016). Epigenetics and aging. Sci. Adv. 2:e1600584. 10.1126/sciadv.1600584 27482540 PMC4966880

[B258] Pandey K. B. Rizvi S. I. (2013). Resveratrol up-regulates the erythrocyte plasma membrane redox system and mitigates oxidation-induced alterations in erythrocytes during aging in humans. Rejuvenation Res. 16, 232–240. 10.1089/rej.2013.1419 23537202

[B259] Pan H. Guan D. Liu X. Li J. Wang L. Wu J.. (2016). SIRT6 safeguards human mesenchymal stem cells from oxidative stress by coactivating NRF2. Cell Res. 26, 190–205. 10.1038/cr.2016.4 26768768 PMC4746611

[B260] Panwar P. Butler G. S. Jamroz A. Azizi P. Overall C. M. Brömme D. (2018). Aging-associated modifications of collagen affect its degradation by matrix metalloproteinases. Matrix Biol. 65, 30–44. 10.1016/j.matbio.2017.06.004 28634008

[B261] Parker L. Mcguckin T. A. Leicht A. S. (2014). Influence of exercise intensity on systemic oxidative stress and antioxidant capacity. Clin. Physiol. Funct. Imaging 34, 377–383. 10.1111/cpf.12108 24283399

[B262] Passos J. F. Nelson G. Wang C. Richter T. Simillion C. Proctor C. J.. (2010). Feedback between p21 and reactive oxygen production is necessary for cell senescence. Mol. Syst. Biol. 6:347. 10.1038/msb.2010.5 20160708 PMC2835567

[B263] Pavel R. Jr. Tabashidze N. Dostal M. Novakova Z. Chvatalova I. Spatova M.. (2011). Genetic, biochemical and environmental factors associated with pregnancy outcomes in newborns from the Czech Republic. Environ. Health Perspect. 119, 265–271. 10.1289/ehp.1002470 20923744 PMC3040616

[B264] Payne B. A. I. Chinnery P. F. (2015). Mitochondrial dysfunction in aging: much progress but many unresolved questions. Biochim. Biophys. Acta 1847, 1347–1353. 10.1016/j.bbabio.2015.05.022 26050973 PMC4580208

[B265] Pearson K. J. Baur J. A. Lewis K. N. Peshkin L. Price N. L. Labinskyy N.. (2008). Resveratrol delays age-related deterioration and mimics transcriptional aspects of dietary restriction without extending life span. Cell Metabol. 8, 157–168. 10.1016/j.cmet.2008.06.011 18599363 PMC2538685

[B266] Pérez V. I. Buffenstein R. Masamsetti V. Leonard S. Salmon A. B. Mele J.. (2009). Protein stability and resistance to oxidative stress are determinants of longevity in the longest-living rodent, the naked mole-rat. Proc. Natl. Acad. Sci. U S A 106, 3059–3064. 10.1073/pnas.0809620106 19223593 PMC2651236

[B267] Pham-Huy L. A. He H. Pham-Huy C. (2008). Free radicals, antioxidants in disease and health. Int. J. Biomed. Sci. 4, 89–96. 23675073 PMC3614697

[B268] Phaniendra A. Jestadi D. B. Periyasamy L. (2015). Free radicals: properties, sources, targets and their implication in various diseases. Indian J. Clin. Biochem. 30, 11–26. 10.1007/s12291-014-0446-0 25646037 PMC4310837

[B269] Pineda-Pampliega J. Herrera-Dueñas A. Mulder E. Aguirre J. I. Höfle U. Verhulst S.. (2020). Antioxidant supplementation slows telomere shortening in free-living white stork chicks. Proc. Biol. Sci. 287:20191917. 10.1098/rspb.2019.1917 31937223 PMC7003462

[B270] Pinti M. Cevenini E. Nasi M. de Biasi S. Salvioli S. Monti D.. (2014). Circulating mitochondrial DNA increases with age and is a familiar trait: implications for “inflamm-aging.”. Eur. J. Immunol. 44, 1552–1562. 10.1002/eji.201343921 24470107

[B271] Poon H. F. Calabrese V. Scapagnini G. Butterfield D. A. (2004). Free radicals and brain aging. Clin. Geriatr. Med. 20, 329–359. 10.1016/j.cger.2004.02.005 15182885

[B272] Popov N. Gil J. (2010). Epigenetic regulation of the INK4B-ARF-INK4a locus: in sickness and in health. Epigenetics 5, 685–690. 10.4161/epi.5.8.12996 20716961 PMC3052884

[B273] Price N. L. Gomes A. P. Ling A. J. Y. Duarte F. V. Martin-Montalvo A. North B. J.. (2012). SIRT1 is required for AMPK activation and the beneficial effects of resveratrol on mitochondrial function. Cell Metabol. 15, 675–690. 10.1016/j.cmet.2012.04.003 22560220 PMC3545644

[B274] Qiu X. Brown K. Hirschey M. D. Verdin E. Chen D. (2010). Calorie restriction reduces oxidative stress by SIRT3-mediated SOD2 activation. Cell Metabol. 12, 662–667. 10.1016/j.cmet.2010.11.015 21109198

[B276] Qiu Z. He Y. Ming H. Lei S. Leng Y. Xia Z.-Y. (2019). Lipopolysaccharide (LPS) aggravates high glucose- and hypoxia/reoxygenation-induced injury through activating ROS-dependent NLRP3 inflammasome-mediated pyroptosis in H9C2 cardiomyocytes. J. Diabetes Res. 2019:8151836. 10.1155/2019/8151836 30911553 PMC6398034

[B277] Raeeszadeh-Sarmazdeh M. Do L. D. Hritz B. G. (2020). Metalloproteinases and their inhibitors: potential for the development of new therapeutics. Cells 9:1313. 10.3390/cells9051313 32466129 PMC7290391

[B278] Raj K. Kaur P. Gupta G. D. Singh S. (2021). Metals associated neurodegeneration in Parkinson’s disease: insight to physiological, pathological mechanisms and management. Neurosci. Lett. 753:135873. 10.1016/j.neulet.2021.135873 33812934

[B279] Ramani S. Pathak A. Dalal V. Paul A. Biswas S. (2020). Oxidative stress in autoimmune diseases: an under dealt malice. Curr. Protein Pept. Sci. 21, 611–621. 10.2174/1389203721666200214111816 32056521

[B280] Ramis M. R. Esteban S. Miralles A. Tan D.-X. Reiter R. J. (2015). Caloric restriction, resveratrol and melatonin: role of SIRT1 and implications for aging and related-diseases. Mech. Ageing Dev. 28, 146–148. 10.1016/j.mad.2015.03.008 25824609

[B281] Ray P. D. Huang B.-W. Tsuji Y. (2012). Reactive oxygen species (ROS) homeostasis and redox regulation in cellular signaling. Cell. Signal. 24, 981–990. 10.1016/j.cellsig.2012.01.008 22286106 PMC3454471

[B282] Raza W. Krachler B. Forsberg B. Sommar J. N. (2020). Health benefits of leisure time and commuting physical activity: a meta-analysis of effects on morbidity. J. Trans. Health 18:100873. 10.1016/j.jth.2020.100873

[B283] Reczek C. R. Chandel N. S. (2015). ROS-dependent signal transduction. Curr. Opin. Cell Biol. 33, 8–13. 10.1016/j.ceb.2014.09.010 25305438 PMC4380867

[B284] Redza-Dutordoir M. Averill-Bates D. A. (2016). Activation of apoptosis signalling pathways by reactive oxygen species. Biochim. Biophys. Acta 1863, 2977–2992. 10.1016/j.bbamcr.2016.09.012 27646922

[B285] Ressler S. Bartkova J. Niederegger H. Bartek J. Scharffetter-Kochanek K. Jansen-Dürr P.. (2006). p16^INK4A^ is a robust *in vivo* biomarker of cellular aging in human skin. Aging Cell 5, 379–389. 10.1111/j.1474-9726.2006.00231.x 16911562

[B286] Rinnerthaler M. Bischof J. Streubel M. K. Trost A. Richter K. (2015). Oxidative stress in aging human skin. Biomolecules 5, 545–589. 10.3390/biom5020545 25906193 PMC4496685

[B287] Rizvi S. I. Maurya P. K. (2007). Alterations in antioxidant enzymes during aging in humans. Mol. Biotechnol. 37, 58–61. 10.1007/s12033-007-0048-7 17914165

[B288] Rodier F. Coppé J.-P. Patil C. K. Hoeijmakers W. A. M. Muñoz D. P. Raza S. R.. (2009). Persistent DNA damage signalling triggers senescence-associated inflammatory cytokine secretion. Nat. Cell Biol. 11, 973–979. 10.1038/ncb1909 19597488 PMC2743561

[B289] Rufini A. Niklison-Chirou M. V. Inoue S. Tomasini R. Harris I. S. Marino A.. (2012). TAp73 depletion accelerates aging through metabolic dysregulation. Genes Dev. 26, 2009–2014. 10.1101/gad.197640.112 22987635 PMC3444727

[B290] Ruiz S. Henschen-Edman A. H. Nagase H. Tenner A. J. (1999). Digestion of C1q collagen-like domain with MMPs-1,-2,-3 and -9 further defines the sequence involved in the stimulation of neutrophil superoxide production. J. Leukoc. Biol. 66, 416–422. 10.1002/jlb.66.3.416 10496311

[B291] Saenen N. D. Martens D. S. Neven K. Y. Alfano R. Bové H. Janssen B. G.. (2019). Air pollution-induced placental alterations: an interplay of oxidative stress, epigenetics and the aging phenotype? Clin. Epigenetics 11:124. 10.1186/s13148-019-0688-z 31530287 PMC6749657

[B292] Salminen A. Ojala J. Kaarniranta K. Haapasalo A. Hiltunen M. Soininen H. (2011). Astrocytes in the aging brain express characteristics of senescence-associated secretory phenotype. Eur. J. Neurosci. 34, 3–11. 10.1111/j.1460-9568.2011.07738.x 21649759

[B293] Schafer M. J. White T. A. Iijima K. Haak A. J. Ligresti G. Atkinson E. J.. (2017). Cellular senescence mediates fibrotic pulmonary disease. Nat. Commun. 8:14532. 10.1038/ncomms14532 28230051 PMC5331226

[B294] Schmidt U. del Monte F. Miyamoto M. I. Matsui T. Gwathmey J. K. Rosenzweig A.. (2000). Restoration of diastolic function in senescent rat hearts through adenoviral gene transfer of sarcoplasmic reticulum Ca^2+^-ATPase. Circulation 101, 790–796. 10.1161/01.cir.101.7.790 10683354

[B295] Scialò F. Fernández-Ayala D. J. Sanz A. (2017). Role of mitochondrial reverse electron transport in ROS signaling: potential roles in health and disease. Front. Physiol. 8:428. 10.3389/fphys.2017.00428 28701960 PMC5486155

[B296] Scialò F. Sriram A. Fernández-Ayala D. Gubina N. Lõhmus M. Nelson G.. (2016). Mitochondrial ROS produced via reverse electron transport extend animal lifespan. Cell Metabol. 23, 725–734. 10.1016/j.cmet.2016.03.009 27076081 PMC4835580

[B298] Selvarani R. Mohammed S. Richardson A. (2021). Effect of rapamycin on aging and age-related diseases—past and future. GeroScience 43, 1135–1158. 10.1007/s11357-020-00274-1 33037985 PMC8190242

[B299] Sentman M.-L. Granström M. Jakobson H. Reaume A. Basu S. Marklund S. L. (2006). Phenotypes of mice lacking extracellular superoxide dismutase and copper- and zinc-containing superoxide dismutase. J. Biol. Chem. 281, 6904–6909. 10.1074/jbc.M510764200 16377630

[B300] Sepidarkish M. Farsi F. Akbari-Fakhrabadi M. Namazi N. Almasi-Hashiani A. Maleki Hagiagha A.. (2019). The effect of vitamin D supplementation on oxidative stress parameters: a systematic review and meta-analysis of clinical trials. Pharmacol. Res. 139, 141–152. 10.1016/j.phrs.2018.11.011 30447293

[B301] Sharov V. S. Galeva N. A. Kanski J. Williams T. D. Schöneich C. (2006). Age-associated tyrosine nitration of rat skeletal muscle glycogen phosphorylase b: characterization by HPLC-nanoelectrospray-Tandem mass spectrometry. Exp. Gerontol. 41, 407–416. 10.1016/j.exger.2006.02.012 16616821

[B302] Simioni C. Zauli G. Martelli A. M. Vitale M. Sacchetti G. Gonelli A.. (2018). Oxidative stress: role of physical exercise and antioxidant nutraceuticals in adulthood and aging. Oncotarget 9, 17181–17198. 10.18632/oncotarget.24729 29682215 PMC5908316

[B303] Slack J. P. Grupp I. L. Dash R. Holder D. Schmidt A. Gerst M. J.. (2001). The enhanced contractility of the phospholamban-deficient mouse heart persists with aging. J. Mol. Cell. Cardiol. 33, 1031–1040. 10.1006/jmcc.2001.1370 11343424

[B304] Smith-Vikos T. Liu Z. Parsons C. Gorospe M. Ferrucci L. Gill T. M.. (2016). A serum miRNA profile of human longevity findings from the baltimore longitudinal study of aging (BLSA). Aging 8, 2971–2987. 10.18632/aging.101106 27824314 PMC5191881

[B305] Sohal R. S. (1976). Metabolic rate and life span. Interdiscip. Top. Gerontol. 9, 25–40.

[B306] Song S. Lam E. W.-F. Tchkonia T. Kirkland J. L. Sun Y. (2020). Senescent cells: emerging targets for human aging and age-related diseases. Trends Biochem. Sci. 45, 578–592. 10.1016/j.tibs.2020.03.008 32531228 PMC7649645

[B307] Srivastava S. (2017). The mitochondrial basis of aging and age-related disorders. Genes 8:398. 10.3390/genes8120398 29257072 PMC5748716

[B308] di Stasi A. M. Mallozzi C. Macchia G. Petrucci T. C. Minetti M. (1999). Peroxynitrite induces tryosine nitration and modulates tyrosine phosphorylation of synaptic proteins. J. Neurochem. 73, 727–735. 10.1046/j.1471-4159.1999.0730727.x 10428070

[B309] Summer R. Shaghaghi H. Schriner D. Roque W. Sales D. Cuevas-Mora K.. (2019). Activation of the mTORC1/PGC-1 axis promotes mitochondrial biogenesis and induces cellular senescence in the lung epithelium. Am. J. Physiol. Lung Cell. Mol. Physiol. 316, L1049–L1060. 10.1152/ajplung.00244.2018 30892080 PMC6620667

[B312] Sun X. Chen W.-D. Wang Y.-D. (2017). DAF-16/FOXO transcription factor in aging and longevity. Front. Pharmacol. 8:548. 10.3389/fphar.2017.00548 28878670 PMC5572328

[B310] Sun L. Sadighi Akha A. A. Miller R. A. Harper J. M. (2009). Life-span extension in mice by preweaning food restriction and by methionine restriction in middle age. J. Gerontol. A. Biol. Sci. Med. Sci. 64, 711–722. 10.1093/gerona/glp051 19414512 PMC2691799

[B311] Sun N. Youle R. J. Finkel T. (2016). The mitochondrial basis of aging. Mol. Cell 61, 654–666. 10.1016/j.molcel.2016.01.028 26942670 PMC4779179

[B313] Su Y. Xu C. Sun Z. Liang Y. Li G. Tong T.. (2019). S100A13 promotes senescence-associated secretory phenotype and cellular senescence via modulation of non-classical secretion of IL-1α. Aging 11, 549–572. 10.18632/aging.101760 30670674 PMC6366962

[B314] Sykora P. Wilson D. M. Bohr V. A. (2012). Repair of persistent strand breaks in the mitochondrial genome. Mech. Ageing Dev. 133, 169–175. 10.1016/j.mad.2011.11.003 22138376 PMC4586262

[B315] Takada Y. Mukhopadhyay A. Kundu G. C. Mahabeleshwar G. H. Singh S. Aggarwal B. B. (2003). Hydrogen peroxide activates NF-κB through tyrosine phosphorylation of IκBα and serine phosphorylation of p65. Evidence for the involvement of IκBα kinase and Syk protein-tyrosine kinase. J. Biol. Chem. 278, 24233–24241. 10.1074/jbc.M212389200 12711606

[B316] Takahashi A. Ohtani N. Yamakoshi K. Iida S.-I. Tahara H. Nakayama K.. (2006). Mitogenic signalling and the p16^INK4a^-Rb pathway cooperate to enforce irreversible cellular senescence. Nat. Cell Biol. 8, 1291–1297. 10.1038/ncb1491 17028578

[B317] Tamm C. Duckworth J. Hermanson O. Ceccatelli S. (2006). High susceptibility of neural stem cells to methylmercury toxicity: effects on cell survival and neuronal differentiation. J. Neurochem. 97, 69–78. 10.1111/j.1471-4159.2006.03718.x 16524380

[B318] Tan B. L. Norhaizan M. E. Liew W.-P.-P. Rahman H. S. (2018). Antioxidant and oxidative stress: a mutual interplay in age-related diseases. Front. Pharmacol. 9:1162. 10.3389/fphar.2018.01162 30405405 PMC6204759

[B319] Tawo R. Pokrzywa W. Kevei É. Akyuz M. E. Balaji V. Adrian S.. (2017). The ubiquitin ligase CHIP integrates proteostasis and aging by regulation of insulin receptor turnover. Cell 169, 470–482. 10.1016/j.cell.2017.04.003 28431247 PMC5406386

[B320] Taylor C. T. Moncada S. (2010). Nitric oxide, cytochrome c oxidase and the cellular response to hypoxia. Arterioscler. Thromb. Vasc. Biol. 30, 643–647. 10.1161/ATVBAHA.108.181628 19713530

[B321] Taylor R. W. Turnbull D. M. (2005). Mitochondrial DNA mutations in human disease. Nat. Rev. Genet. 6, 389–402. 10.1038/nrg1606 15861210 PMC1762815

[B323] Tennen R. I. Chua K. F. (2011). Chromatin regulation and genome maintenance by mammalian SIRT6. Trends Biochem. Sci. 36, 39–46. 10.1016/j.tibs.2010.07.009 20729089 PMC2991557

[B324] Tisi A. Feligioni M. Passacantando M. Ciancaglini M. Maccarone R. (2021). The impact of oxidative stress on blood-retinal barrier physiology in age-related macular degeneration. Cells 10, 1–21. 10.3390/cells10010064 33406612 PMC7823525

[B325] Tong N. Jin R. Zhou Z. Wu X. (2019). Involvement of microRNA-34a in age-related susceptibility to oxidative stress in ARPE-19 cells by targeting the silent mating type information regulation 2 homolog 1/p66shc pathway: implications for age-related macular degeneration. Front. Aging Neurosci. 11:137. 10.3389/fnagi.2019.00137 31249522 PMC6584679

[B326] Tosato M. Zamboni V. Ferrini A. Cesari M. (2007). The aging process and potential interventions to extend life expectancy. Clin. Interv. Aging 2, 401–412. 18044191 PMC2685272

[B327] Trifunovic A. Hansson A. Wredenberg A. Rovio A. T. Dufour E. Khvorostov I.. (2005). Somatic mtDNA mutations cause aging phenotypes without affecting reactive oxygen species production. Proc. Natl. Acad. Sci. U S A 102, 17993–17998. 10.1073/pnas.0508886102 16332961 PMC1312403

[B239] United Nations (2019). World Population Ageing 2019 Highlights. Available online at: https://www.un-ilibrary.org/content/books/9789210045537. Accessed September 25, 2021.

[B328] Valacchi G. Fortino V. Bocci V. (2005). The dual action of ozone on the skin. Br. J. Dermatol. 153, 1096–1100. 10.1111/j.1365-2133.2005.06939.x 16307642

[B329] Valavanidis A. Vlachogianni T. Fiotakis K. (2012). Recent scientific advances on the free radical and oxidative stress theory of ageing. Dietary supplements of antioxidants or caloric restriction for reversing ageing? Pharmakeftiki 24, 2–12.

[B330] Valavanidis A. Vlachogianni T. Rallis M. (2015). The controversy for the validity of the free radical or oxidative stress theory of ageing: recent scientific evidence for oxidative damage at molecular level, animals and ageing population - Based studies. Pharmakeftiki 27, 31–50.

[B331] Valko M. Leibfritz D. Moncol J. Cronin M. T. D. Mazur M. Telser J. (2007). Free radicals and antioxidants in normal physiological functions and human disease. Int. J. Biochem. Cell Biol. 39, 44–84. 10.1016/j.biocel.2006.07.001 16978905

[B85] van Deursen J. M. (2014). The role of senescent cells in ageing. Nature 509, 439–446. 10.1038/nature13193 24848057 PMC4214092

[B332] Vasto S. Barera A. Rizzo C. di Carlo M. Caruso C. Panotopoulos G. (2014). Mediterranean diet and longevity: an example of nutraceuticals? Curr. Vasc. Pharmacol. 12, 735–738. 10.2174/1570161111666131219111818 24350926

[B333] Velarde M. C. Flynn J. M. Day N. U. Melov S. Campisi J. (2012). Mitochondrial oxidative stress caused by Sod2 deficiency promotes cellular senescence and aging phenotypes in the skin. Aging 4, 3–12. 10.18632/aging.100423 22278880 PMC3292901

[B334] Ventura A. Luzi L. Pacini S. Baldari C. T. Pelicci P. G. (2002). The p66Shc longevity gene is silenced through epigenetic modifications of an alternative promoter. J. Biol. Chem. 277, 22370–22376. 10.1074/jbc.M200280200 11948181

[B335] Victorelli S. Lagnado A. Halim J. Moore W. Talbot D. Barrett K.. (2019). Senescent human melanocytes drive skin ageing via paracrine telomere dysfunction. EMBO J. 38:e101982. 10.15252/embj.2019101982 31633821 PMC6885734

[B336] Viña J. (2019). The free radical theory of frailty: mechanisms and opportunities for interventions to promote successful aging. Free Radic. Biol. Med. 134, 690–694. 10.1016/j.freeradbiomed.2019.01.045 30735838

[B372] von Zglinicki T. (2002). Oxidative stress shortens telomeres. Trends Biochem. Sci. 27, 339–344. 10.1016/s0968-0004(02)02110-2 12114022

[B337] Wadhwa R. Gupta R. Maurya P. K. (2018). Oxidative stress and accelerated aging in neurodegenerative and neuropsychiatric disorder. Curr. Pharm. Des. 24, 4711–4725. 10.2174/1381612825666190115121018 30644343

[B338] Wajapeyee N. Serra R. W. Zhu X. Mahalingam M. Green M. R. (2008). Oncogenic BRAF induces senescence and apoptosis through pathways mediated by the secreted protein IGFBP7. Cell 132, 363–374. 10.1016/j.cell.2007.12.032 18267069 PMC2266096

[B339] Waldman T. Kinzler K. W. Vogelstein B. (1995). p21 is necessary for the p53-mediated G1 arrest in human cancer cells. Cancer Res. 55, 5187–5190. 7585571

[B340] Wallerath T. Deckert G. Ternes T. Anderson H. Li H. Witte K.. (2002). Resveratrol, a polyphenolic phytoalexin present in red wine, enhances expression and activity of endothelial nitric oxide synthase. Circulation 106, 1652–1658. 10.1161/01.cir.0000029925.18593.5c 12270858

[B341] Walters M. S. De B. P. Salit J. Buro-Auriemma L. J. Wilson T. Rogalski A. M.. (2014). Smoking accelerates aging of the small airway epithelium. Respir. Res. 15:94. 10.1186/s12931-014-0094-1 25248511 PMC4189169

[B343] Wang C. Jurk D. Maddick M. Nelson G. Martin-ruiz C. von Zglinicki T. (2009). DNA damage response and cellular senescence in tissues of aging mice. Aging Cell 8, 311–323. 10.1111/j.1474-9726.2009.00481.x 19627270

[B345] Wang D. Malo D. Hekimi S. (2010). Elevated mitochondrial reactive oxygen species generation affects the immune response via hypoxia-inducible factor-1α in long-lived Mclk1^+/–^ mouse mutants. J. Immunol. 184, 582–590. 10.4049/jimmunol.0902352 20007531

[B346] Wang L. Sesso H. D. Glynn R. J. Christen W. G. Bubes V. Manson J. E.. (2014). Vitamin E and C supplementation and risk of cancer in men: posttrial follow-up in the Physicians’ health study II randomized trial. Am. J. Clin. Nutr. 100, 915–923. 10.3945/ajcn.114.085480 25008853 PMC4135500

[B342] Wang C.-H. Wei Y.-H. (2020). Roles of mitochondrial sirtuins in mitochondrial function, redox homeostasis, insulin resistance and type 2 diabetes. Int. J. Mol. Sci. 21, 1–18. 10.3390/ijms21155266 32722262 PMC7432223

[B348] Wang Y. Yang J.-Q. Hong T.-T. Sun Y.-H. Huang H.-L. Chen F.. (2020). RTN4B-mediated suppression of Sirtuin 2 activity ameliorates β-amyloid pathology and cognitive impairment in Alzheimer’s disease mouse model. Aging Cell 19:e13194. 10.1111/acel.13194 32700357 PMC7431833

[B347] Wang X. Zhou Y. Gao Q. Ping D. Wang Y. Wu W.. (2020). The role of exosomal microRNAs and oxidative stress in neurodegenerative diseases. Oxid. Med. Cell. Longev. 2020:3232869. 10.1155/2020/3232869 33193999 PMC7641266

[B349] Warraich U.-E.-A. Hussain F. Kayani H. U. R. (2020). Aging - Oxidative stress, antioxidants and computational modeling. Heliyon 6:e04107. 10.1016/j.heliyon.2020.e04107 32509998 PMC7264715

[B350] Waters D. W. Schuliga M. Pathinayake P. S. Wei L. Tan H.-Y. Blokland K. E. C.. (2021). A senescence bystander effect in human lung fibroblasts. Biomedicines 9:1162. 10.3390/biomedicines9091162 34572347 PMC8470192

[B351] Weidinger A. Kozlov A. V. (2015). Biological activities of reactive oxygen and nitrogen species: oxidative stress versus signal transduction. Biomolecules 5, 472–484. 10.3390/biom5020472 25884116 PMC4496681

[B352] White M. J. McArthur K. Metcalf D. Lane R. M. Cambier J. C. Herold M. J.. (2014). Apoptotic caspases suppress mtDNA-induced STING-mediated type i IFN production. Cell 159, 1549–1562. 10.1016/j.cell.2014.11.036 25525874 PMC4520319

[B353] Wiggins K. A. Parry A. J. Cassidy L. D. Humphry M. Webster S. J. Goodall J. C.. (2019). IL-1α cleavage by inflammatory caspases of the noncanonical inflammasome controls the senescence-associated secretory phenotype. Aging Cell 18:e12946. 10.1111/acel.12946 30916891 PMC6516163

[B354] Wilkinson J. E. Burmeister L. Brooks S. V. Chan C.-C. Friedline S. Harrison D. E.. (2012). Rapamycin slows aging in mice. Aging Cell 11, 675–682. 10.1111/j.1474-9726.2012.00832.x 22587563 PMC3434687

[B355] Wong R. H. X. Howe P. R. C. Buckley J. D. Coates A. M. Kunz I. Berry N. M. (2011). Acute resveratrol supplementation improves flow-mediated dilatation in overweight/obese individuals with mildly elevated blood pressure. Nutr. Metab. Cardiovasc. Dis. 21, 851–856. 10.1016/j.numecd.2010.03.003 20674311

[B356] Wood S. H. Dam S. V. Craig T. Tacutu R. O’Toole A. Merry B. J.. (2015). Transcriptome analysis in calorie-restricted rats implicates epigenetic and post-translational mechanisms in neuroprotection and aging. Genome Biol. 16:285. 10.1186/s13059-015-0847-2 26694192 PMC4699360

[B357] Xiao X. Xu M. Yu H. Wang L. Li X. Rak J.. (2021). Mesenchymal stem cell-derived small extracellular vesicles mitigate oxidative stress-induced senescence in endothelial cells via regulation of miR-146a/Src. Signal Transduct. Target. Ther. 6:354. 10.1038/s41392-021-00765-3 34675187 PMC8531331

[B358] Xiong Y. Xiong Y. Zhang H. Zhao Y. Han K. Zhang J.. (2021). hPMSCs-derived exosomal miRNA-21 protects against aging-related oxidative damage of CD4^+^ T cells by targeting the PTEN/PI3K-Nrf2 axis. Front. Immunol. 12:780897. 10.3389/fimmu.2021.780897 34887868 PMC8649962

[B360] Xu S. Cai Y. Wei Y. (2014). mTOR signaling from cellular senescence to organismal aging. Aging Dis. 5, 263–273. 10.14336/AD.2014.0500263 25110610 PMC4113516

[B359] Xu D. Tahara H. (2013). The role of exosomes and microRNAs in senescence and aging. Adv. Drug Delivery Rev. 65, 368–375. 10.1016/j.addr.2012.07.010 22820533

[B363] Xu T. Sun L. Shen X. Chen Y. Yin Y. Zhang J.. (2019). NADPH oxidase 2-mediated NLRP1 inflammasome activation involves in neuronal senescence in hippocampal neurons *in vitro*. Int. Immunopharmacol. 69, 60–70. 10.1016/j.intimp.2019.01.025 30677569

[B361] Xu S. Wu W. Huang H. Huang R. Xie L. Su A.. (2019). The p53/miRNAs/Ccna2 pathway serves as a novel regulator of cellular senescence: Complement of the canonical p53/p21 pathway. Aging Cell 18:e12918. 10.1111/acel.12918 30848072 PMC6516184

[B362] Xu S. Zhong M. Zhang L. Wang Y. Zhou Z. Hao Y.. (2009). Overexpression of Tfam protects mitochondria against β-amyloid-induced oxidative damage in SH-SY5Y cells. FEBS J. 276, 3800–3809. 10.1111/j.1742-4658.2009.07094.x 19496804

[B364] Yalcinkaya T. Uzilday B. Ozgur R. Turkan I. Mano J. (2019). Lipid peroxidation-derived reactive carbonyl species (RCS): their interaction with ROS and cellular redox during environmental stresses. Environ. Exp. Botany 165, 139–149. 10.1016/j.envexpbot.2019.06.004

[B365] Yang D. Elner S. G. Bian Z.-M. Till G. O. Petty H. R. Elner V. M. (2007). Pro-inflammatory cytokines increase reactive oxygen species through mitochondria and NADPH oxidase in cultured RPE cells. Exp. Eye Res. 85, 462–472. 10.1016/j.exer.2007.06.013 17765224 PMC2094037

[B367] Yang R. Chen J. Zhang J. Qin R. Wang R. Qiu Y.. (2020). 1,25-Dihydroxyvitamin D protects against age-related osteoporosis by a novel VDR-Ez h2-p16 signal axis. Aging Cell 19:e13095. 10.1111/acel.13095 31880094 PMC6996957

[B366] Yang H. Zhang W. Pan H. Feldser H. G. Lainez E. Miller C.. (2012). SIRT1 activators suppress inflammatory responses through promotion of p65 deacetylation and inhibition of NF-κB activity. PLoS One 7:e46364. 10.1371/journal.pone.0046364 23029496 PMC3460821

[B368] Yermilov V. Rubio J. Becchi M. Friesen M. D. Pignatelli B. Ohshima H.. (1995). Formation of 8-nitroguanine by the reaction of guanine with peroxynitrite *in vitro*. Carcinogenesis 16, 2045–2050. 10.1093/carcin/16.9.2045 7554052

[B369] Yousefzadeh M. J. Zhao J. Bukata C. Wade E. A. McGowan S. J. Angelini L. A.. (2020). Tissue specificity of senescent cell accumulation during physiologic and accelerated aging of mice. Aging Cell 19:e13094. 10.1111/acel.13094 31981461 PMC7059165

[B370] Yu A. Q. Wang J. Zhou X. J. Chen K. Y. Cao Y. D. Wang Z. X.. (2020). Senescent cell-secreted netrin-1 modulates aging-related disorders by recruiting sympathetic fibers. Front. Aging Neurosci. 12:507140. 10.3389/fnagi.2020.507140 33390926 PMC7772213

[B371] Yun J. Finkel T. (2014). Mitohormesis. Cell Metabol. 19, 757–766. 10.1016/j.cmet.2014.01.011 24561260 PMC4016106

[B373] Zhang Y. Zhang J. Wang S. (2021). The role of rapamycin in healthspan extension via the delay of organ aging. Ageing Res. Rev. 70:101376. 10.1016/j.arr.2021.101376 34089901

[B374] Zhao L. Cao J. Hu K. He X. Yun D. Tong T.. (2020). Sirtuins and their biological relevance in aging and age-related diseases. Aging Dis. 11, 927–945. 10.14336/AD.2019.0820 32765955 PMC7390530

[B375] Zhao X. Ren X. Zhu R. Luo Z. Ren B. (2016). Zinc oxide nanoparticles induce oxidative DNA damage and ROS-triggered mitochondria-mediated apoptosis in zebrafish embryos. Aquat. Toxicol. 180, 56–70. 10.1016/j.aquatox.2016.09.013 27658222

[B376] Zhou W. Tian D. He J. Wang Y. Zhang L. Cui L.. (2016). Repeated PM2.5 exposure inhibits BEAS-2B cell P53 expression through ROS-Akt-DNMT3B pathway-mediated promoter hypermethylation. Oncotarget 7, 20691–20703. 10.18632/oncotarget.7842 26942697 PMC4991485

[B377] Zhu Y. Tchkonia T. Pirtskhalava T. Gower A. C. Ding H. Giorgadze N.. (2015). The achilles’ heel of senescent cells: from transcriptome to senolytic drugs. Aging Cell 14, 644–658. 10.1111/acel.12344 25754370 PMC4531078

[B378] Zorov D. B. Juhaszova M. Sollott S. J. (2014). Mitochondrial reactive oxygen species (ROS) and ROS-induced ROS release. Physiol. Rev. 94, 909–950. 10.1152/physrev.00026.2013 24987008 PMC4101632

[B379] Zsurka G. Peeva V. Kotlyar A. Kunz W. S. (2018). Is there still any role for oxidative stress in mitochondrial DNA-dependent aging? Genes 9:175. 10.3390/genes9040175 29561808 PMC5924517

[B380] Zuo L. He F. Sergakis G. G. Koozehchian M. S. Stimpfl J. N. Rong Y.. (2014). Interrelated role of cigarette smoking, oxidative stress and immune response in COPD and corresponding treatments. Am. J. Physiol. Lung Cell. Mol. Physiol. 307, L205–L218. 10.1152/ajplung.00330.2013 24879054

